# A probable koala from the Oligocene of central Australia provides insights into early diprotodontian evolution

**DOI:** 10.1038/s41598-023-41471-0

**Published:** 2023-09-04

**Authors:** Arthur I. Crichton, Robin M. D. Beck, Aidan M. C. Couzens, Trevor H. Worthy, Aaron B. Camens, Gavin J. Prideaux

**Affiliations:** 1 College of Science and Engineering, Flinders University, Bedford Park, 5042 Australia; 2 School of Science, Engineering and Environment, University of Salford, Salford, England; 3 Department of Ecology and Evolutionary Biology, University of California, Los Angeles, CA 90095 USA

**Keywords:** Palaeontology, Taxonomy

## Abstract

Diprotodontians are the morphologically and ecologically most diverse order of marsupials. However, an approximately 30-million-year gap in the Australian terrestrial vertebrate fossil record means that the first half of diprotodontian evolution is unknown. Fossil taxa from immediately either side of this gap are therefore critical for reconstructing the early evolution of the order. Here we report the likely oldest-known koala relatives (Phascolarctidae), from the late Oligocene Pwerte Marnte Marnte Local Fauna (central Australia). These include coeval species of *Madakoala* and *Nimiokoala*, as well as a new probable koala (?Phascolarctidae). The new taxon, *Lumakoala blackae* gen. et sp. nov., was comparable in size to the smallest-known phascolarctids, with body-mass estimates of 2.2–2.6 kg. Its bunoselenodont upper molars retain the primitive metatherian condition of a continuous centrocrista, and distinct stylar cusps B and D which lacked occlusion with the hypoconid. This structural arrangement: (1) suggests a morphocline within Phascolarctidae from bunoselenodonty to selenodonty; and (2) better clarifies the evolutionary transitions between molar morphologies within Vombatomorphia. We hypothesize that the molar form of *Lumakoala blackae* approximates the ancestral condition of the suborder Vombatiformes. Furthermore, it provides a plausible link between diprotodontians and the putative polydolopimorphians *Chulpasia jimthorselli* and *Thylacotinga bartholomaii* from the early Eocene Tingamarra Local Fauna (eastern Australia), which we infer as having molar morphologies consistent with stem diprotodontians.

## Introduction

**Figure 1:**
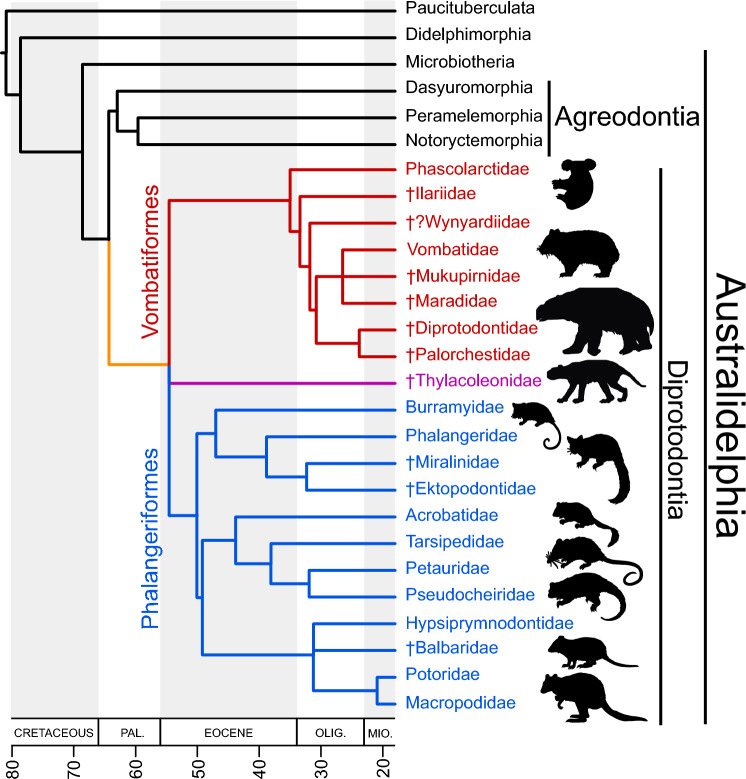
Cladistic reconstruction of diprotodontian interrelationships. Relationships and divergence estimates for extant lineages follow Duchêne et al.^6^. Those of extinct lineages follow Beck et al.^5^, Travouillon et al.^102^, and Crichton et al.^38,39^. The divergence estimates for extinct vombatiforms and phalangeroids are speculatory.

Australia is renowned for the uniqueness of its biota, particularly its marsupials. This remarkable endemism is due mainly to their Gondwanan ancestry^1,2^, and long isolation following the continent’s separation from Antarctica around 45 million years ago (mya)^3,4^. Much of the morphological and ecological diversity of Australian marsupials is manifested within the order Diprotodontia, which comprises two suborders of primarily herbivorous taxa—Vombatiformes (wombats, koalas and extinct relatives) and Phalangerida (possums and kangaroos)—as well as Thylacoleonidae (marsupial ‘lions’; Diprotodontia incertae sedis^5^) (Fig. 1). Molecular-clock analyses suggest that Diprotodontia diverged from Agreodontia (Dasyuromorphia + Peramelemorphia + Notoryctemorphia) sometime between the latest Cretaceous and the earliest middle Eocene^5–14^. This is important from a biogeographic standpoint because it implies that diprotodontians had diverged before the final separation of Australia and Antarctica. Yet, no definitive fossil diprotodontians from prior to the late Oligocene are known, with the possible exception of diprotodontian-like tarsals from the early or middle Eocene of southern Argentina^15^.

Just two Australian fossil assemblages dating between 110 and 25 mya have yielded mammals^16,17^, leaving a massive gap spanning virtually the entire window of Australian marsupial origins^18,19^. In this window, taxon descriptions have only been published for metatherians (marsupials and stem-relatives) from the early Eocene (55 mya) Tingamarra Local Fauna (LF) of south-eastern Queensland, all on the basis of isolated dental, periotic and tarsal specimens^17,20–27^. These elements have been interpreted to lack unequivocal synapomorphies linking them with ordinal-level marsupial taxa known from the Australian late Oligocene^1,9,17,19,21^. Of these early Eocene taxa, only *Djarthia murgonensis* has been referred to Australidelphia (Australian marsupials + Microbiotheria of South America)^22^. Other Tingamarra LF marsupials have been argued to bear greater affinity with bunodont polydolopimorphians from South America^23,25,27^.

By the time the Australian terrestrial mammal record recommences, near the end of the Oligocene (c. 25 mya), most modern diprotodontian families are represented^21,28,29^. The distinctiveness of their dental and skeletal morphologies, as well as molecular disparity among modern representatives, suggest long pre-Miocene divergence intervals^5,21,30,31^. Each family is characterized by a specialized molar dentition that occupies a unique region in a morphospace that includes selenodont (multiple crescents), bunodont (bulbous cusps) and bilophodont (two transverse crests) forms. Identifying homologies between these molar architectures, and reconstructing the transformations that led to their acquisition, has long been considered important to resolving the broader problem of diprotodontian interrelationships^5,30,32–40^. The vombatiform radiation has been of particular interest; the extant representatives (koalas and wombats) capture but a small amount of the total morphological diversity known to have existed.

Koalas (Phascolarctidae) have selenodont molars, characterized by serial crescentic crests that function as leaf cutters during mastication (e.g.,^41–43^). Winge^44^ considered the molar form of the modern koala to be the least specialized among known diprotodontians, being only slightly modified from that of the archetypal metatherian condition. This view was accepted by Archer^32^, who suggested that selenodonty in diprotodontians could have arisen directly from a peramelemorphian (bandicoot) type morphology. This was based largely on the observation that modern koala and bandicoot molars lack a continuous crest (centrocrista) connecting the paracone and metacone, with the postparacrista and premetacrista instead linked to a cusp in the stylar cusp C and D positions, respectively^32^. It has since become evident that this morphology is derived within Peramelemorphia^45–47^; nevertheless, the view that vombatiforms transitioned from selenodonty to lophodonty has been widely supported^30,33–35^.

**Figure 2:**
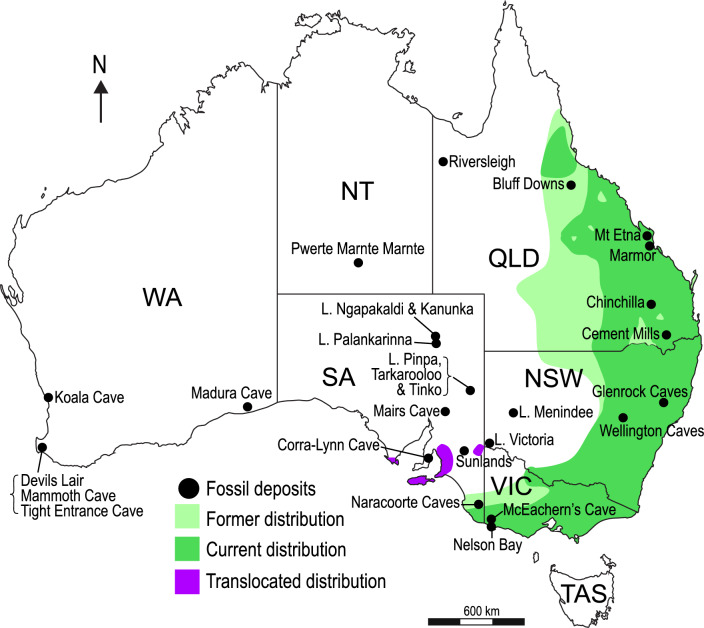
Map of Australia depicting phascolarctid occurrences, modified from Price^59^ and Black et al.^54^. Fossil sites that have yielded phascolarctid remains are indicated with black circles. *Phascolarctos cinereus* former historical distribution is indicated in light green; current distribution in dark green; and translocated distribution in purple.

This study reports newly discovered fossils of koalas, and probable koalas, from the Pwerte Marnte Marnte deposit, southern Northern Territory, the likely-oldest Oligocene terrestrial mammal bearing site on the continent^29,48^ (Fig. 2). We describe a new genus and species characterized by a distinctive molar morphology that helps clarify evolutionary patterns within Diprotodontia, and may shed light on the phylogenetic affinities of enigmatic early Eocene metatherians from Australia. We also report the presence of two other koala species from the site.

### Systematic palaeontology

Infraclass Marsupialia Illiger, 1811

Order Diprotodontia Owen, 1877

Suborder Vombatiformes Woodburne, 1984

Infraorder ?Phascolarctomorphia Aplin & Archer, 1987

Family ?Phascolarctidae Owen, 1839

**Figure 3:**
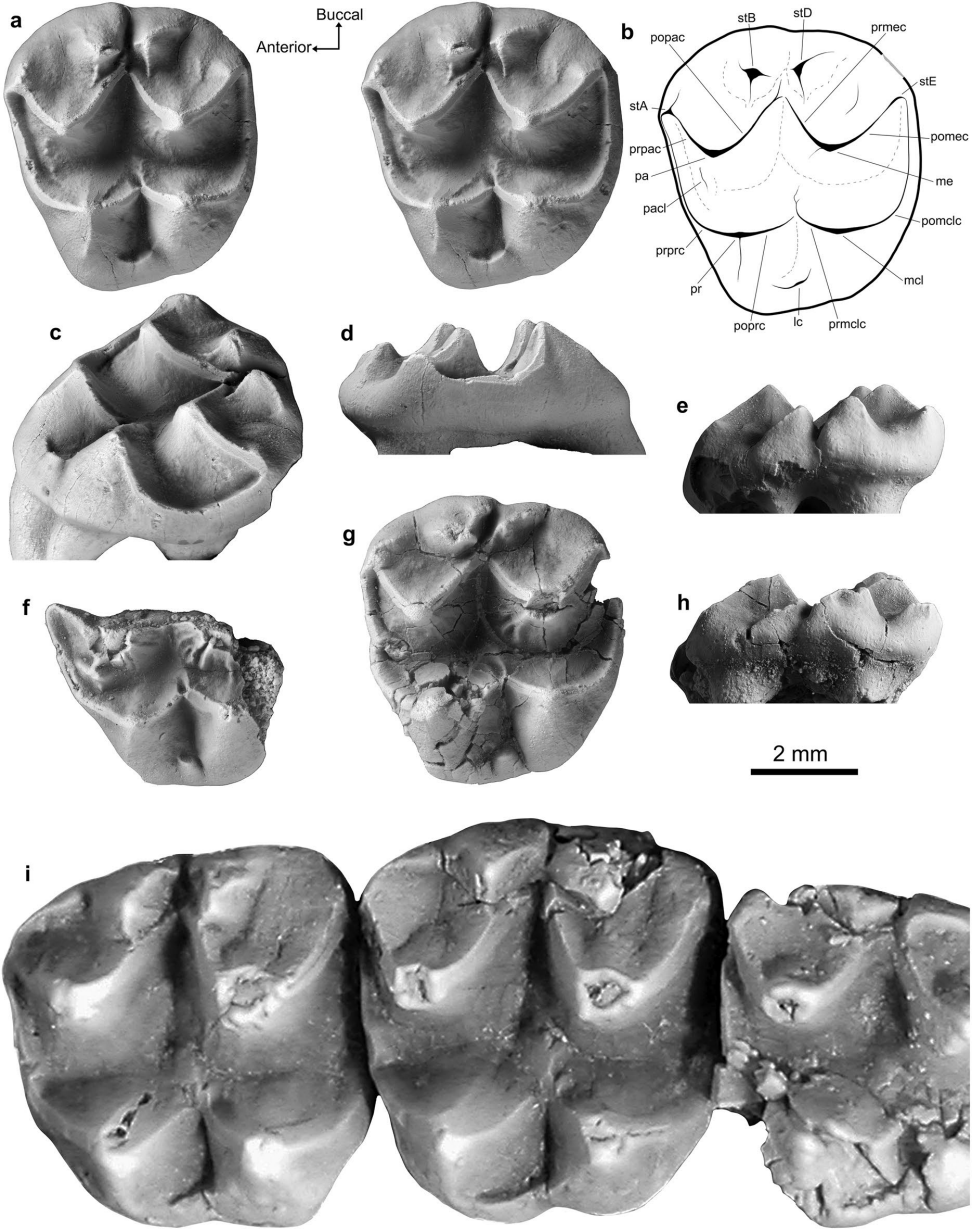
*Lumakoala blackae* gen. et sp. nov. upper molars. Left M 2? or 3 (Holotype, NTM P12012): (**a**) stereo occlusal view, with (**b**) an annotated line drawing; (**c**) posterolingually offset occlusal view; (**d**) anterior view; and (**e**) buccal view. Anterolingual half of LM1 (Paratype, NTM P12014) in (**f**) occlusal view. Left M ?2 or 3 (Paratype, NTM P12013) in (**g**) occlusal and (**h**) buccal views. *Priscakoala lucyturnbullae* left M1–M3 (Holotype, QM F20203), adapted from Black et al.^58^. The orientation arrows (Anterior, Buccal) relate only to the specimens in occlusion view. *lc* lingual cingulum, *me* metacone, *mcl* metaconule, *pa* paracone, *pacl* paraconule, *pomclc* postmetaconulecrista, *pomec* postmetacrista, *popac* postparacrista, *poprc* postprotocrista, *pr* protocone, *prmclc* premetaconulecrista, *prmec* premetacrista, *prpac* preparacrista, *prprc* preprotocrista, *stA* stylar cusp A, *stB* stylar cusp B, *stD* stylar cusp D, *stE* stylar cusp E.

*Lumakoala blackae* gen. et sp. nov. (Fig. 3)

**Holotype**. NTM P12012, left M2 or M3 (Fig. 3a–e).

**Paratypes**. NTM P12013, left M2 or M3 (Fig. 3g–h); NTM P12014, anterolingual half of left M1 (Fig. 3f).

**Locality**. Pwerte Marnte Marnte fossil site (24°21′ S 133°43′ E), deriving from an unnamed geological formation on the southern flank of the James Range, Northern Territory, Australia.

**Local Fauna, and Age**. Initial biochronological assessments have suggested that the Pwerte Marnte Marnte LF is late Oligocene age^38,39,48^, and may predate the oldest late Oligocene assemblages from the Etadunna and Namba Formations of the southern Lake Eyre Basin, perhaps corresponding to an as-yet-unnamed land mammal age immediately preceding the Etadunnan^29,48^.

**Genus Etymology**. *Luma* is Latin for ‘thorn’, in reference to the morphology of stylar cusps B and D, and their distinction from the postparacrista and premetacrista, respectively. The gender of the genus is feminine.

**Species Etymology**. Named for Karen Black, whose research has greatly extended our understanding of fossil phascolarctids and other vombatiforms. 

**Genus Diagnosis**. As for the species until another is recognized.

**Specific Diagnosis**. A new species that appears most similar in upper molar morphology to members of the Phascolarctidae, with: a deep, U-shaped longitudinal valley between the primary buccal and lingual cusps, rather than being shallow and V-shaped as in selenodont vombatomorphians; and a paraconule on M1 (Fig. 3a–h).

*Lumakoala blackae* is relatively small compared to known phascolarctids, with upper molars that fall within the lower end of the size range of the smallest species of *Litokoala*. The taxon differs from all phascolarctids in having an M2 with: stylar cusps B and D that are somewhat conical and more structurally independent from the postparacrista and premetacrista, respectively; a postparacrista that continues beyond its junction with the premetacrista towards stylar cusp D; a wider stylar shelf, wherein the junction between the premetacrista and postparacrista is roughly mid-width between the buccal margin and the apices of the paracone and metacone; and more rounded buccal and posterior margins in occlusal view, leading to a generally rounder outline.

*Lumakoala blackae* differs from all other phascolarctids, unless otherwise noted, in having: proportionately wider molars relative to length (except *Litokoala garyjohnstoni*); more buccally situated protocone and metaconule apices, with a steeper buccal face of the protocone and metaconule compared with the lingual face (except *L. garyjohnstoni*); stylar cusp B taller than stylar cusp A on M2 (except *Priscakoala lucyturnbullae*); a small cuspate paraconule that extends up the anterolingual face of the paracone on M1 (except species of *Phascolarctos*); lacking a neometaconule (except *Priscakoala lucyturnbullae*); and lacking a hypertrophied anterobuccal terminus of the preprotocrista on M1 (except *Priscakoala lucyturnbullae,* species of *Madakoala* and *Perikoala*).

*Lumakoala blackae* is most similar to *Priscakoala lucyturnbullae,* sharing: simple enamel surface ornamentation without a neometaconule; stylar cusp B taller than stylar cusp A; cuspate stylar cusps B and D; and a wide stylar shelf. In addition to the differences identified with respect to all phascolarctids, *L. blackae* differs from *Priscakoala lucyturnbullae* in having: fine crenulations on the lingual faces of the paracone and metacone; a paraconule on M1; a stylar cusp A that is considerably larger, being similar in height to stylar cusp D; a prominent crista that descends from the apex of the metacone down the anterolingual face; a short and sometimes cuspate rather than elongate lingual cingulum; and a metaconule reduced relative to protocone on M2, resulting in an anterior moiety of markedly greater width than the posterior moiety.

**Description**. The upper molars of *Lumakoala blackae* (Fig. 3a–h) are low crowned, with fine crenulations on the lingual faces of the paracone and metacone. The molars have bunoselenodont crown morphology composed of four principal cusps, with the two anterior cusps representing the paracone (buccal) and protocone (lingual), and the posterior two representing the metacone (buccal) and metaconule (lingual). Two additional, somewhat conical cusps are present, buccal to the postparacrista and premetacrista, which are referred to as stylar cusps B and D, respectively (see Supplementary 1 for discussion of stylar cusp homologies). The cusp in the anterobuccal corner of the tooth, at the terminus of the preparacrista, is referred to as stylar cusp A.

Specimens NTM P12012 and NTM P12013 are both considered to represent M2, or possibly M3, because: tooth width tapers anteroposteriorly; and there is a prominent buccolingually extensive depression spanning the anterior and posterior faces, deriving from contact with the neighboring upper molars. Based on comparison with the molars of *Priscakoala lucyturnbullae*, NTM P12012 and NTM P12013 are thought to most likely represent M2, rather than M3 because, in the latter, stylar cusps B and D are markedly reduced. The specimens attributed to the M2 position are trapezoidal from occlusal view with rounded tooth corners, deriving from a strongly convex buccal margin and a lingual margin that curves posterobuccally. The molar proportions taper from anterior to posterior and buccal to lingual (Fig. 3). The anterior width of NTM P12012 is greater than posterior width by 7%, and greater than length by 12% (Table S1). The specimen NTM P12013 is thoroughly fractured, and consequently has slightly inflated dimensions (Table S1). On NTM P12013, there is also damage to the postprotocrista, the apex of the metacone, and the posterobuccal corner of the tooth. Greatest wear is present along the edges of the cristae. Description of the M2 morphology is based primarily on the holotype, NTM P12012, owing to its better preservation.

The metacone is the largest cusp, and the metaconule is the smallest (Fig. 3). The buccal basins of the paracone and metacone are only slightly concave as they slope from the apices to meet the boundaries of the stylar cusps. The buccal margin is open between each of (1) the stylar cusps A and B, and (2) stylar cusp D and the terminus of the postmetacrista (Fig. 3). The paracone apex is positioned slightly anterior relative to the protocone apex, and slightly buccal relative to the metacone apex. A linear preparacrista descends anterobuccally to a slightly raised cuspate structure in the anterobuccal corner of the tooth, representing stylar cusp A. Two cristae descend from the stylar cusp A: one posterobuccally, terminating anterior to stylar cusp B; and the other anterolingually, that is then continuous with the preprotocrista. The latter is not hypertrophied anteriorly into a cuspate structure (referred to as a parastyle in Black et al.^49^). A linear postparacrista descends posterobuccally, meeting the premetacrista proximate to the lingual extremity of stylar cusps B and D. The postparacrista continues weakly beyond its juncture with the premetacrista towards stylar cusp D before terminating. In NTM P12013, the postparacrista is continuous with a crista descending anterolingually from stylar cusp D, which, together, close off stylar cusp B. The postparacrista and premetacrista are structurally independent from stylar cusps B and D, respectively.

Stylar cusps B and D are somewhat conical;stylar cusp B is the tallest, being only c. 20% shorter than the paracone (Fig. 3e–h). Several short cristae descend from the apices of stylar cusps B and D, differentially expressed between NTM P12012 and NTM P12013 (Fig. 3). In NTM P12012, the most prominent crista descending from the apex of stylar cusp B is oriented anterolingually, weakly parallel to the postparacrista. From stylar cusp D, one crista is oriented posterolingually, parallel to the premetacrista, and a second is oriented posteriorly. By comparison, on NTM P12013, the most prominent crista descending from stylar cusp B is oriented posterobuccally; and from stylar cusp D, one is oriented anterolingually and another posterobuccally, together forming a crest. No structures link the stylar cusps. On NTM P12012, a faint ridge descends the metacone buccal basin anterior to the postmetacrista. The postmetacrista descends posterobuccally from the apex of the metacone, towards the posterobuccal tooth corner where it then arches ventrally, becoming continuous with the postmetaconulecrista. There is a slight swelling at the posterobuccal terminus of the postmetacrista, which represents stylar cusp E. The paracone and metacone are each somewhat conical in profile, wherein the lingual and buccal faces are similarly sloped. A faint crista descends from the apex of the metacone down the anterolingual face, terminating before the longitudinal basin. On NTM P12013, the crista is more prominent and bifurcates midway down the anterolingual face of the metacone.

The protocone is positioned slightly more posteriorly than the paracone, and slightly more lingual than the metaconule (Fig. 3). A crescentic preprotocrista descends anterobuccally from the apex of the protocone, curving as it spans the anterior margin of the tooth. On specimens NTM P12012 and NTM P12013, a faint ridge ascends the anterolingual face of the paracone, representing an incipient paraconule. On NTM P12013, there is also a short crista posterolingual to the apex of the paracone in the longitudinal valley that forms a slight swelling as it intercepts the preprotocrista (Fig. 3). This structure is considered a protostyle, the absence of which on NTM P12012 may be intraspecific variation. A linear postprotocrista descends posteriorly, intercepting the premetaconulecrista at the transverse valley. The premetaconulecrista continues beyond its junction with the postprotocrista before terminating. The postprotocrista and premetaconulecrista are each only slightly buccally inclined, together forming a relatively wide lingual angle of 115 degrees, compared to the 75-degree angle formed by the postparacrista and premetacrista. The metaconule apex is slightly posterior to the metacone apex. A crescentic postmetaconulecrista curves posterobuccally along the posterior margin of the tooth. The buccal faces of the protocone and metaconule are steeper than the lingual faces, with the latter representing roughly a third of the tooth width from occlusal view. A short lingual cingulum closes off the transverse valley at the lingual margin between the protocone and metaconule. On NTM P12013, the lingual cingulum is cuspate.

An anterolingual half of an upper molar (NTM P12014) has generally similar morphology to NTM P12012 and NTM P12013. This molar fragment is considered to represent an M1 on the basis that: the posterior moiety seems to have been wider than the anterior moiety; the paraconule is relatively more prominent; the depression on the anterior face deriving from the point of the contact with (presumably) the posterior face of P3, is small and circular (rather than buccolingually extensive). The paraconule is cuspate, positioned on the anterolingual face of the paracone. In common with specimen NTM P12014, two prominent cristae, aligned parallel one another, descend the anterolingual face of the metacone (Fig. 3). The placement of these two cristae relative to the metacone mirrors that of the paraconule relative to the paracone.

**Figure 4:**
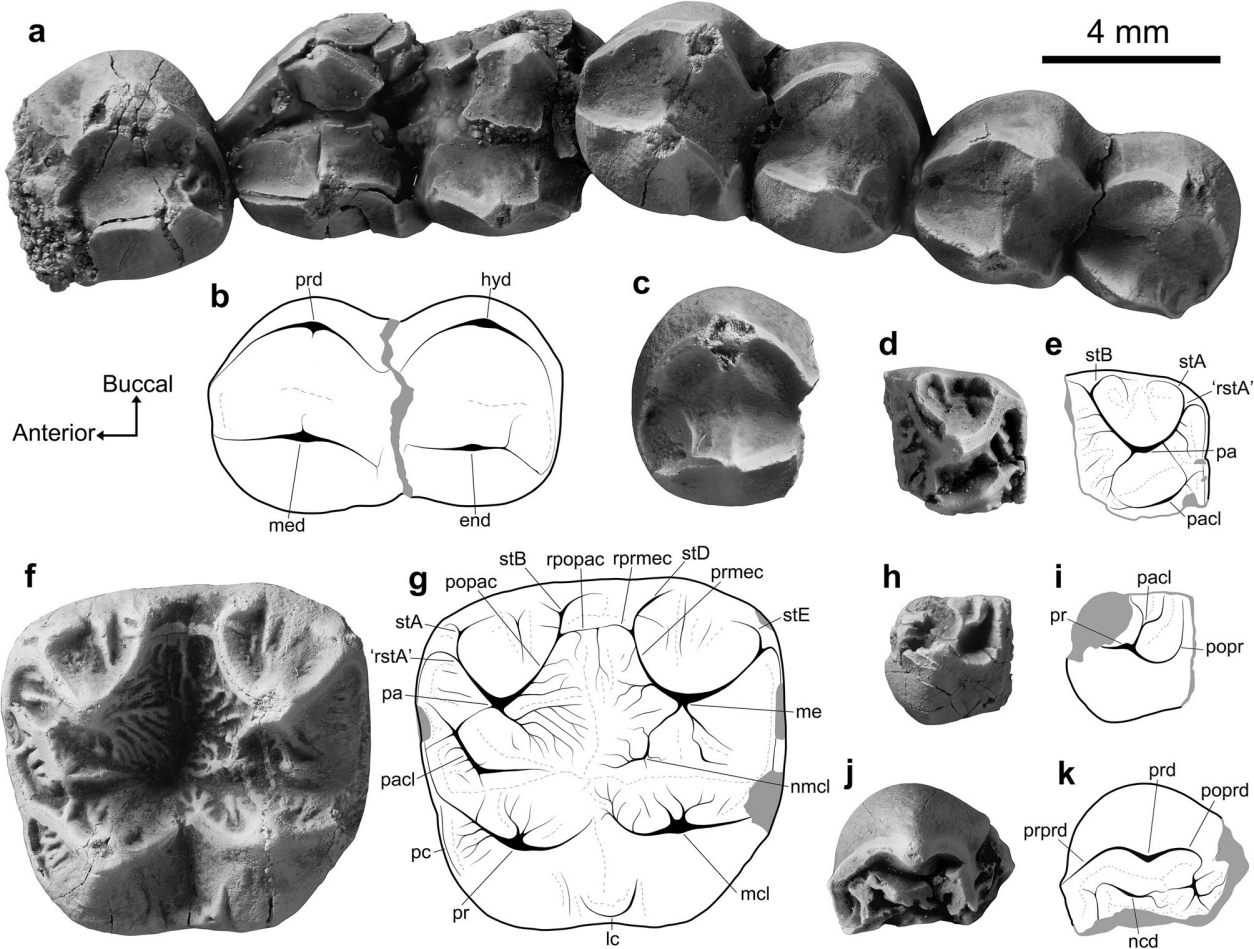
Specimens of *Madakoala* sp. cf. *M*. *devisi* and *Nimiokoala* sp. indet. from the Pwerte Marnte Marnte site, with annotated line drawings. *Madakoala* sp. cf. *M*. *devisi*: (**a**, **b**) partial right lower molar row preserving m1–m4 (NTM P12015); (**c**) left m3 talonid (NTM P12016); (**d**, **e**) paracone from right M 2? (NTM P12017); (**f**, **g**) left M1 (NTM P14005). *Nimiokoala* sp. indet.: (**h**,** i**) left protocone (NTM P12018); (**j**, **k**) right protoconid (NTM P12019). The orientation arrows (Anterior, Buccal) relate to the right lower molar (**a**, **b**, **j**, **k**), and left upper molar (**f**, **g**, **h**, **i**), specimens. *end* entoconid, *hyd* hypoconid, *lc* lingual cingulum, *mcl* metaconule, *me* metacone, *med* metaconid, *ncd* neomorphic cuspid, *nmcl* neometaconule, *pa* paracone, *pacl* paraconule, *pc* precingulum, *popac* postparacrista, *popr* postprotocrista, *poprd* postprotocristid, *pr* protocone, *prd* protoconid, *prmec* premetacrista, *prprd* preprotocristid, *rpopac* remnant postparacrista, *rprmec* remnant postmetacrista, *rstA* remnant stylar cusp A, *stA* Stylar cusp A, s*tB* stylar cusp B, *stD* Stylar cusp D, *stE* stylar cusp E.

***Madakoala*** **sp. cf.** ***M. devisi.*** Lower molar specimens of the largest phascolarctid in the Pwerte Marnte Marnte LF compare closely to those of *Madakoala devisi* in size (Table S2, Supplementary 2) and morphology (Fig. 4a–c; and Supplementary 3 for descriptions). Species of *Madakoala* are otherwise known from the late Oligocene Namba and Etadunna Formations in the southern Lake Eyre Basin^50,51^. We note that the lower molar specimens (NTM P12015 and NTM P12016) are more similar in morphology to *M. devisi* than the upper molar specimens (Fig. 4d–g; NTM P14005; NTM P12017), with the latter also similar to species of *Perikoala* (see Supplementary 3). It is possible that these specimens do not derive from the same taxon. The occurrence of representatives from both genera in a single assemblage has been reported in Ditjimanka LF of the Etadunna Formation^50,51^. At this stage, we consider it most parsimonious to treat these specimens as belonging to a single species allied to *M. devisi*.

***Nimiokoala*** **sp. indet.** Two molar fragments (NTM P12017, NTM P12018: Fig. 4h–k) compare best with species of *Nimiokoala*, though both are too fragmentary for systematic assessment beyond generic referral (Supplementary 4). The only other late Oligocene record of the genus, represented by a partial dentary (SAMA P19952) from the Namba Formation^52^, is not yet referred to a species. The type species, *Nimiokoala greystanesi*, is known from the Early to Middle Miocene of Riversleigh, north-west Queensland^52–56^.

**Results of phylogenetic analyses.** Parsimony analyses with all taxa included, and ordering of states where morphoclines were inferred, generated a strict consensus of 58 most parsimonious trees, each of 498 steps (Fig. S4, Supplementary 7). The strict consensus tree had a consistency index of 0.39 and a retention index of 0.77. Our maximum parsimony (strict consensus) and undated Bayesian analyses (majority rule consensus topology of the post-burnin trees) recovered similar topologies to the equivalent analyses of Crichton et al.^39^ (Figs. S2–S5, Supplementary 7). This reflects that relatively few modifications were made to the morphological characters of Crichton et al.^39^. In our unconstrained Bayesian analysis, *Lumakoala blackae* was weakly supported (BPP = 0.61) as the sister taxon to a moderately supported Phascolarctidae + Vombatomorphia (BPP = 0.82); whereas, under maximum parsimony, it formed a polytomy (BS = 0.88) with *Cercartetus lepidus*, Thylacoleonidae, and a weakly supported Phascolarctidae + Vombatomorphia (BS = 14%). The placement of *L. blackae* outside of Phascolarctidae + Vombatomorphia was supported by two character-transformations: stC not linked to paracone on M2 (C.I. = 0.33: char. 41); and metacone not linked to stD on M2 (C.I. = 0.50: char. 47). *Thylacotinga bartholomaii* consistently formed the sister taxon to Diprotodontia (BBP = 0.97; BS = 0.87).

The alternative hypothesis for the placement of *Lumakoala blackae*, tested by constraining it into a clade with phascolarctids, yielded 52 most parsimonious trees, each with a length of 499 steps, differing from that of the unconstrained topology by only one step. This was associated with a non-significant (P =  > 0.05) difference in character state distribution, as indicated by the one-sided Templeton test (P = 0.28–0.43). As such, the hypothesis that *Lumakoala blackae* is a phascolarctid cannot be rejected. Strict consensus of the most parsimonious trees from the constrained analysis recovered Phascolarctidae as a polytomy composed of *Lumakoala blackae*, *Priscakoala lucyturnbullae*, and a clade comprising the remaining koala taxa (Fig. S5, Supplementary 7).

## Discussion

The discovery of phascolarctids in the Pwerte Marnte Marnte fossil site provides the first record of koalas from the Northern Territory in both the historical and fossil records. Assuming *Lumakoala blackae* is indeed a phascolarctid, the site preserves three koala species, representing the equal highest number recorded from a single deposit, along with Camel Sputum Site from the early Miocene (Faunal Zone B) of Riversleigh^57,58^. Surprisingly, the fossil sites from the late Oligocene (Faunal Zone A) of Riversleigh have not yielded any phascolarctids^55,56^. From the late Oligocene Namba and Etadunna Formations of central Australia, phascolarctids are represented in most local faunas, with a single taxon in most, and two in some^50^. It has been suggested that the high early Miocene koala diversity from Riversleigh may correlate with greater plant diversity in these presumably rainforest communities^54^. The high phascolarctid diversity in the Pwerte Marnte Marnte LF may thus also indicate the presence of high plant diversity in a wooded paleoenvironment.

If *Lumakoala blackae* gen. et sp. nov. is a phascolarctid, it is the tenth genus and among the geologically oldest-known. Its discovery further emphasizes the well-noted^54,59^ phascolarctid turnover from the late Oligocene (*Lumakoala, Madakoala*, *Perikoala*, *Nimiokoala* and *Litokoala*) to early Miocene (*Nimiokoala* and *Litokoala*, *Priscakoala*). *Lumakoala blackae* was relatively small, yielding body mass estimates of 2.2–2.6 kg (Table S3, Supplementary 8) based on regression equations^60^ of M2 and M3 length. This falls well within the size range of the modern brushtail possum *Trichosurus vulpecula* (1.5–4.5 kg^61^), and is markedly smaller than other reportedly plesiomorphic phascolarctids (e.g., species of *Priscakoala*, *Madakoala*, *Perikoala*, *Koobor* and *Invictokoala*), all of which fall within the size range of the modern koala species (see^54^: 4.1–13.5 kg^62^). Indeed, *L. blackae* is among the smallest-known phascolarctids, together with species of *Litokoala* (measurements in Table S1, Supplementary 2).

**Figure 5:**
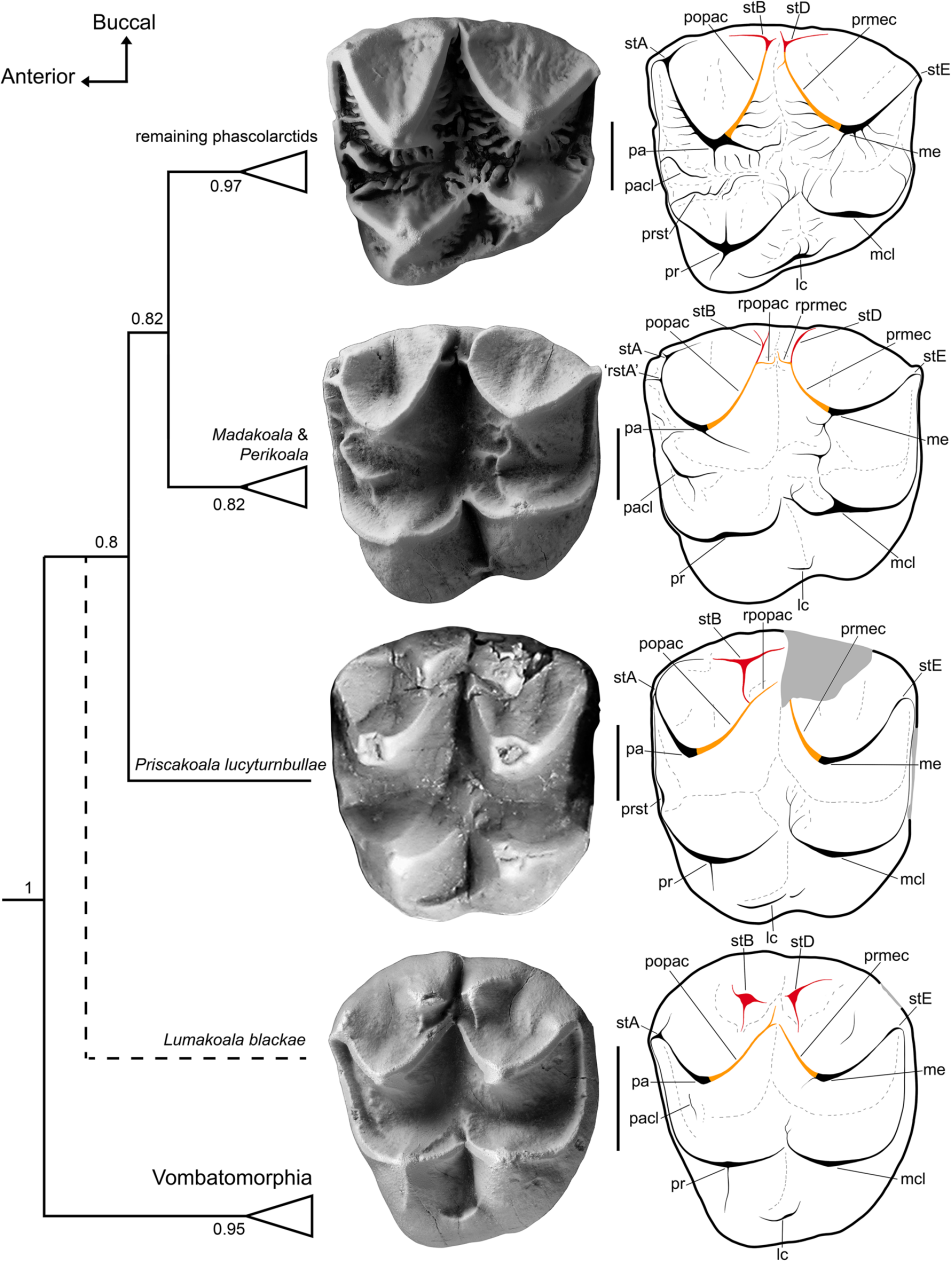
Concatenated phascolarctid interrelationships, contextualized by upper molars from representative taxa in occlusal view, with annotated line drawings. From top to bottom: *Phascolarctos cinereus* (M2, FUR 095); *Madakoala devisi* (M2, FUR unregistered; field number BC 20–112); *Priscakoala lucyturnbullae* (M2, QM F20203) adapted from Black et al.^58^; and the probable phascolarctid *Lumakoala blackae*, gen. et sp. nov. (Left M2 or 3, NTM P12012). Topology based on undated Bayesian analysis, presented as a majority rule consensus, with numbers at nodes representing Bayesian posterior probabilities (Fig. S3, Supplementary 7 for complete tree). *Lumakoala blackae* was excluded from this analysis; its hypothesized placement within Phascolarctidae is indicated by a dashed line. *lc* lingual cingulum, *me* metacone, *mcl* metaconule, *pa* paracone, *pacl* paraconule, *popac* postparacrista, *pr* protocone, *prmec* postmetacrista, *prst* protostyle, *rpopac* remnant postparacrista, *rprmec* remnant postmetacrista, *rstA* remnant stylar cusp A, s*tA* stylar cusp A, *stB* stylar cusp B, *stD* stylar cusp D, *stE* stylar cusp E. Scale bar equals 2 mm.

Unlike the selenodont molars of unequivocal phascolarctids, those of *L. blackae* have a wide stylar shelf with large, conical stylar cusps B and D, that are unconnected from the postparacrista and premetacrista, respectively (Fig. 5). From a functional standpoint, this crown morphology, and the associated wear pattern, suggest no occlusion between the stylar cusps and the hypoconid during the transverse (shearing) phase of the power stroke. Instead, stylar cusps B and D would have overhung the buccal face of the lower molars, presumably abetting puncture-crushing during the initial vertical phase. Direct occlusion between the stylar cusps and the hypoconid may, nonetheless, have been possible as an artifact of high tooth wear later in life history. More generally, the low crown height and poorly developed enamel crenulations are consistent with adaptation for relatively soft foods. We hypothesize that *L. blackae* had a primarily plant-based diet, although it may have opportunistically consumed insects like many extant possums^62^.

The presence of this bunoselenodont molar morphology in a diprotodontian marsupial is phylogenetically significant. This is because the structural independence of stylar cusps B and D, and a continuous centrocrista (postparacrista + premetacrista), are unambiguous symplesiomorphies of the other australidelphian orders (Microbiotheria, Peramelemorphia, Dasyuromorphia and Notoryctemorphia), as well as in Didelphimorphia and many other metatherian clades (e.g., see ^5^).

Our unconstrained Bayesian phylogenetic analyses imply that *L*. *blackae* is a stem vombatiform, sister to Phascolarctidae + Vombatomorphia, though with weak support (BBP = 0.61; for complete trees, see Fig. S2, Supplementary 7). Inclusion of *L. blackae* within the analyses also resulted in lower support for monophyly of Phascolarctidae and Vombatiformes (Figs. S2 and S3, Supplementary 7), reflecting that the morphology known of the new taxon blurs the boundaries of these clades. This poor resolution may not be surprising given that *L. blackae* could only be scored for 18% of the morphological characters, as it is known only from upper molars. Furthermore, there are no obvious upper molar synapomorphies of Phascolarctidae, with the possible exception of a paraconule on M1, which is present in all phascolarctids except *Priscakoala lucyturnbullae*^58^. Based on a Templeton test, the tree length of the topology in which *L. blackae* is constrained to form a clade with definitive phascolarctids is not significantly worse than the unconstrained topology in which *L. blackae* is a stem vombatiform (Figs. S4 and S5, Supplementary 7). At this stage, we consider it most appropriate to consider *L. blackae* a probable phascolarctid.

Within Phascolarctidae, the upper molar morphology of *L. blackae* is most similar to that of *P. lucyturnbullae*. Both are low crowned, have prominent stylar cusps, simple enamel surface ornamentation, and lack a neometaconule (Fig. 5). *Priscakoala lucyturnbullae* has previously been viewed^58^ as the most plesiomorphic phascolarctid known to date because it: lacks a paraconule and neometaconule on M1; has a weakly expressed protostylid on m1; and has simple, uncrenulated, selenodont molars. On this basis, the molars of *L. blackae* could be considered slightly more derived than those of *P. lucyturnbullae* because a paraconule is present on M1 and also (although only weakly developed) on M2. Nevertheless, we argue that the upper molar morphology of *L. blackae* is, actually more plesiomorphic than that of *P. lucyturnbullae*, because it extends phascolarctid molar morphospace from variations on a strict selenodont form, to bunoselenodonty. From the more plesiomorphic to more derived end of this proposed phascolarctid morphocline, we observe: a reduction of the stylar shelf; integration of stylar cusps B and D into the postparacrista and premetacrista, respectively; reduction of stylar cusp B relative to stylar cusps A and D; and increasing obliquity of the angle formed at the postprotocrista–premetaconulecrista juncture (Fig. 5).

**Figure 6:**
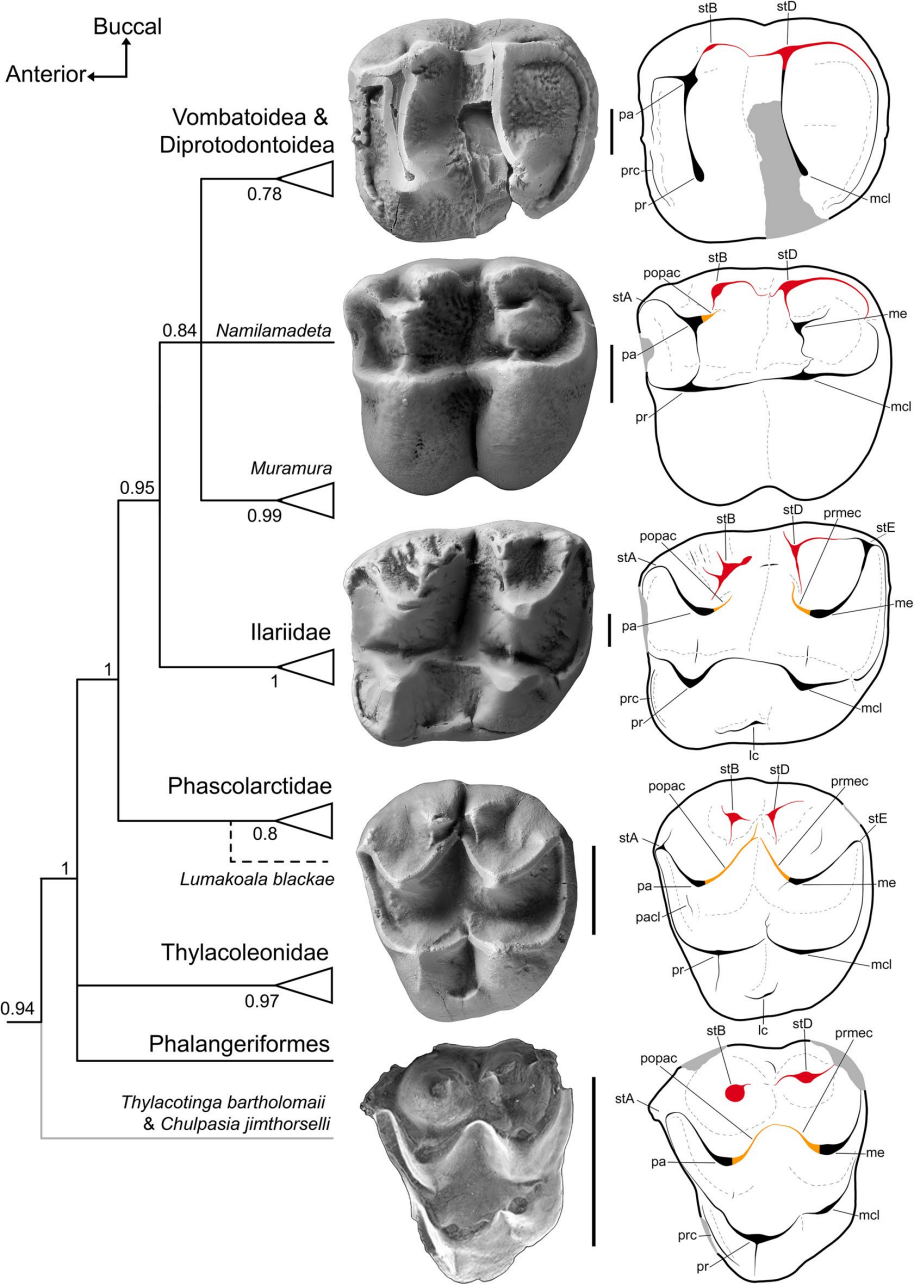
Vombatiform interrelationships, contextualized by upper molars from representative taxa in occlusal view, with annotated line drawings. From top to bottom: the diprotodontoid, *Raemeotherium yatkolai* (RM1, SAMA P43060: photograph mirrored); ?wynyardiid, *Namilamadeta crassirostrum* (LM1, cast of AR 9745); ilariid, *Ilaria illumidens* (M1, SAMA P43055); and the probable phascolarctid, *Lumakoala blackae*, gen. et sp. nov., (isolated left M2 or 3, NTM P12012). The putative polydolopimorphian *Chulpasia jimthorselli* is represented at the bottom by an upper molar (left M1 or 2, QM F50411). Concatenated topology based on undated Bayesian analysis, presented as a majority rule consensus, with numbers at nodes representing Bayesian posterior probabilities (Fig. S3, Supplementary 7 for complete tree). *Lumakoala blackae* was excluded from this analysis; its hypothesized placement within Phascolarctidae is indicated by a dashed line. *lc* lingual cingulum, *mcl* metaconule, *me* metacone, *pa* paracone, *pacl* paraconule, *popac* postparacrista, *pr* protocone, *prc* precingulum, *prmec* premetacrista, *prst* protostyle, *stA* stylar cusp A, *stB* stylar cusp B, *stD* stylar cusp D, *stE* stylar cusp E. Scale bar equals 2 mm.

The molar morphology of *Lumakoala blackae* is also intermediate between that of the otherwise basal selenodont vombatomorphians and the archetypal metatherian condition (Fig. 6). In particular, it confirms that some selenodont vombatomorphians retain remnants of a continuous centrocrista, lingual to large cuspate stylar cusps B and D on M1; these include the ilariids *Kuterintja ngama* (see fig. 2^63^) and *Ilaria illumidens* (Fig. 6), as well as the ?wynyardiid *Muramura pinpensis*. The atrophy of these structures occurs in a stepwise manner, from posterior to anterior along the molar row. The postparacrista and premetacrista are lost in more derived vombatiforms (Fig. 6).

**Figure 7:**
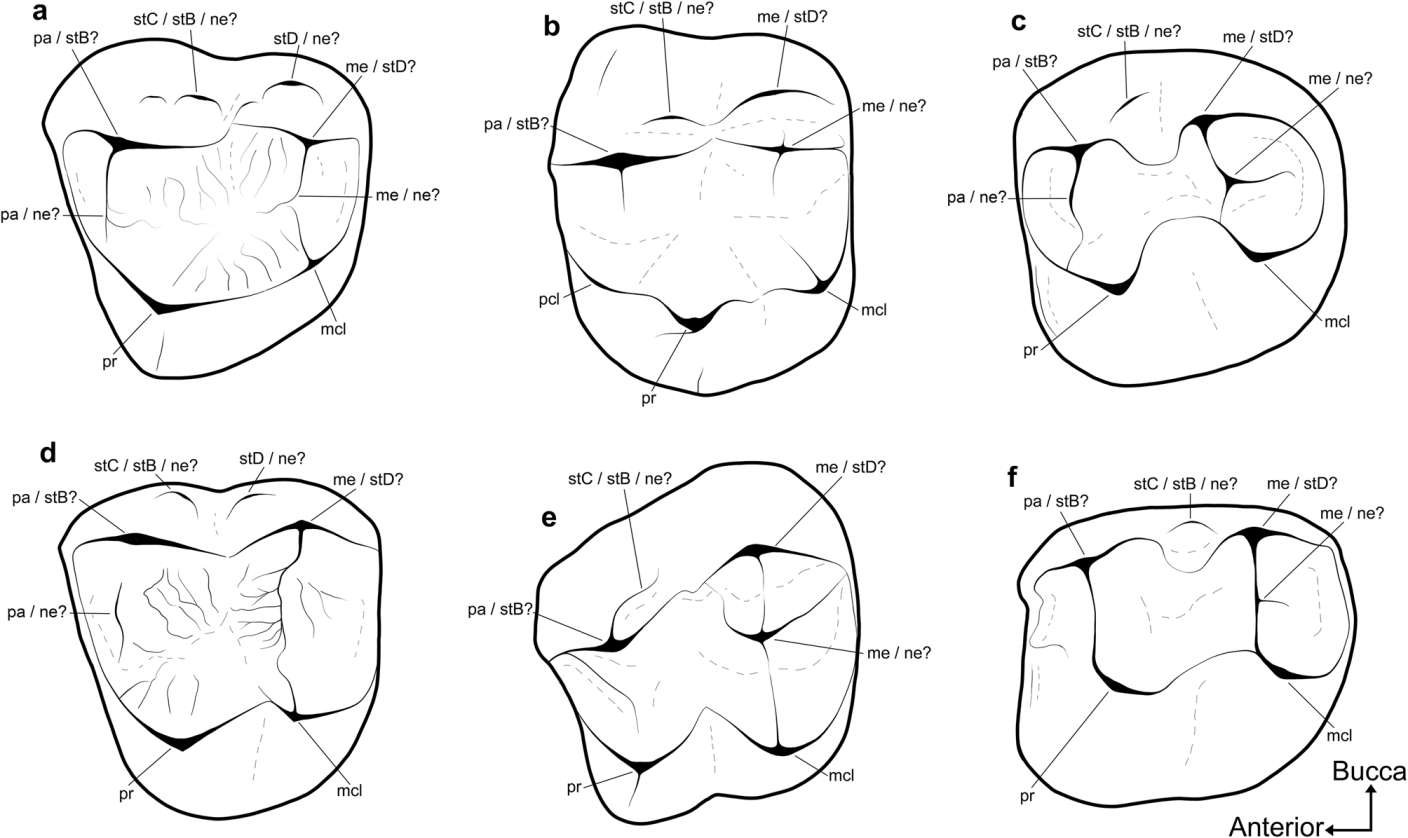
Annotated line drawings of upper molars from representative non-vombatiform diprotodontians, illustrating the ambiguity concerning homology of their buccal structures. The thylacoleonid (**a**) *Lekaneleo roskellyae* (RM1, mirrored; QM F23442); phalangeroids (**b**) *Burramys wakefieldi* (LM1; SAMA P40931), and (**e**) *Durudawiri inusitatus* (RM1, mirrored; QM F31468); macropodoids (**c**) *Palaeopotorous priscus* (RM1 or M2, mirrored; NMV P157547), and (**f**) *Nambaroo tarrinyeri* (LM1 or M2, 157528); and miminipossumid? (**d**) gen. et sp. indet. (LM1 or M2, Ditjimanka Local Fauna; SAMA P19856). *mcl* metaconule, *me* metacone, *ne* neomorphic structure, *pa* paracone, *pcl* protoconule, *pr* protocone, *stB* stylar cusp B, *stC* stylar cusp C, *stD* stylar cusp D. Not to scale.

It is less clear how metatherian stylar cusp homologies translate to the somewhat bunolophodont molars of thylacoleonids and most early phalangeriforms (Fig. 7; see^5,36,38,64^ and references therein). Thylacoleonids have been interpreted, by some, as retaining the symplesiomorphic condition of a continuous centrocrista and a distinct stylar shelf^38,65,66^. It has also been suggested that that the small buccal-most structures on the M1 of thylacoleonids are actually neomorphic, and that the primary buccal cusps, traditionally referred to as the paracone and metacone, are actually stylar in origin^5^. Many early macropodoids and some phalangeroids bear a small cuspate ridge on the posterobuccal face of the paracone on M1^38^, which has traditionally been identified as stylar cusp C^36,67^. However, many early phalangeriforms also bear central cusps on their upper molars, most commonly on the posterior moiety of M1. The latter configuration has led several authors to flag the possibility that the cusps traditionally referred to as the paracone and metacone may instead represent stylar cusps B and D, respectively^68,69^, and that any subsidiary structures buccal to them are neomorphic^5^.

In light of the stepwise morphocline observed between the molar topographies of phascolarctid genera (Fig. 5), and more generally between the vombatiform families (Fig. 6), we posit that the bunoselenodont molar morphology of *L. blackae* is close to the ancestral condition for Vombatiformes; this is congruent with the stem vombatiform position for this taxon in the unconstrained Bayesian analysis. It is noteworthy that body mass estimates for *Lumakoala blackae* (2.2–2.6 kg: Table S3, Supplementary 8) also fall within bounds predicted for the ancestor of Vombatiformes (1–5.5 kg)^30^. With the exception of the relative prominence of the metaconule, this morphology probably does not differ markedly from the ancestral condition for Diprotodontia. These observations imply that crown clade Diprotodontia (Vombatiformes + Phalangeriformes + Thylacoleonidae) retained the symplesiomorphic australidelphian condition of structurally independent stylar cusps B and D, and a continuous centrocrista. If correct, the reduction and/or integration of stylar cusps B and D into proximate structures has occurred convergently in several diprotodontian lineages as they evolved distinct, specialized dentitions.

Most molecular divergence estimates imply that diprotodontians were present when the early Eocene sediments near Murgon were deposited (e.g.,^6–12^), but none have been identified from the associated Tingamarra LF^21^. The plesiomorphic molar form of *L. blackae* provides an unexpected link between the molar morphologies of the Tingamarran taxa *Chulpasia jimthorselli* and *Thylacotinga bartholomaii* and the earliest undoubted diprotodontians (Fig. 6). Correspondingly, these Tingamarran taxa are plausible antecedent diprotodontians. Key molar attributes possessed by these Tingamarran taxa include: prominent and structurally independent stylar cusps B and D; a continuous centrocrista; a small but distinct metaconule; a posterobuccal corner that forms an angle > 60°; and a paracone and protocone that are subequal in size on M2 (see Supplementary 9 for further comparisons).

*Thylacotinga bartholomaii* and *Chulpasia jimthorselli* have been reconstructed as omnivores^23,25^, with body mass estimates of 3.2 kg and 0.2 kg, respectively^27^; the former being considerably larger than other mammals described to date from the Tingamarra LF. It has been suggested that these chulpasiines became extinct during the Palaeogene^23^. When included in our primarily vombatiform phylogenetic analyses, *Thylacotinga bartholomaii* was consistently recovered with strong support as the sister taxon to Diprotodontia (Fig. 6), to the exclusion of the representative agreodontians (for complete trees, Figs. S2–S5 Supplementary 7). A link between these Tingamarran taxa and definitive diprotodontians is attractive in its simplicity, supporting continuity between the early Eocene and late Oligocene Australian marsupial faunas, but we recognize that this may be an artifact of the very poor early Paleogene fossil record from Australia.

*Thylacotinga bartholomaii* and *Chulpasia jimthorselli*, together with its congener *C. mattaueri* from the late Paleocene or early Eocene of Peru, have been placed in the subfamily Chulpasiinae and referred to the primarily South American order Polydolopimorphia^23^. However, there remains contention concerning the relationship of *C. jimthorselli* to *C. mattaueri*, and thus the biogeographic inferences that can be drawn^70,71^. This is because all three taxa are known from isolated molars^23,25,72^, and have been grouped together on the basis of generalized bunodont features that are known to be highly homoplastic within Metatheria^2,73,74^. A formal phylogenetic analysis has yet to be published that tests their relationships to each other and to other bunodont and non-bunodont marsupialiforms. As such, a diprotodontian affinity for *Thylacotinga bartholomaii* and *Chulpasia jimthorselli* may not necessitate a pan-Gondwanan distribution for the australidelphian order (as would be implied if these two taxa are closely related to South American taxa). Nevertheless, it is worth recognizing that Lorente et al.^15^ described isolated australidelphian marsupial tarsals, from the La Barda locality (which now appears to be ~ 43.5 mya or younger^75^) in southern Argentina, that fell within crown Diprotodontia in their phylogenetic analysis. Putative chulpasiines have not been described from La Barda or a second site with a taxonomically similar mammalian fauna, Laguna Fria^76^, and it is unclear whether the La Barda tarsals belong to any of the taxa from the site known from dental remains^2,15^.

It has also been hypothesized, though contentiously, that Diprotodontia may be allied to Polydolopimorphia (the order to which Sige et al.^23^, referred Chulpasiinae) together deriving from a microbiotherian-like ancestor^15,77–83^. However, the phylogenetic affinities of polydolopimorphians are quite controversial, both with respect to their ordinal relationships and their monophyly as a single clade^2,74^. Furthermore, phylogenomic and retroposon insertion data provides compelling evidence that Microbiotheria is sister to the modern Australian marsupial radiation as whole^6,14,84^ rather than just Diprotodontia specifically.

Regardless of whether or not *Thylacotinga* and *Chulpasia* are in fact early diprotodontians, we hypothesize that their bunodont molar morphology—well suited to an omnivorous diet—is probably very close to the ancestral diprotodontian condition. Unlike tribosphenic metatherians, the power stroke in most extant diprotodontians is divided into a vertical phase and a transverse phase, with the latter accomplished by drawing the lower molars of the occluding side medially across the upper molars^85^. Interestingly, the upper molar specimens of *T. bartholomaii* show distinct wear facets on the crests linking stB and stD, presumably due to occlusion with the hypoconid, indicating a strong transverse component to the masticatory stroke. Indeed, much of the required change in molar morphology from the form of *Thylacotinga* and *Chulpasia*, to that of *Lumakoala* and other selenodont diprotodontians, can be explained as adaptations for facilitating a stronger transverse component for specialized herbivory. The formation of continuous transverse crests between the paracone and metacone, and stylar cusps B and D, respectively, increases shearing blade length; whereas loss of the link between the postparacrista and premetacrista allows the hypoconid to move further buccally, forming a larger crushing basin. These changes, alongside cranial adaptions enabling a larger transverse masticatory stroke, probably represented crucial steps in the transition from insectivorous to herbivorous diets in diprotodontian evolution.

## Methods

The phascolarctid specimens reported in this study were recovered from ~ 2 tonnes of limestone quarried from the Pwerte Marnte Marnte fossil beds on expeditions in 2014 and 2020, led by A. Couzens and A. Crichton, respectively. The fossiliferous rock was processed during 2020–2022 in the Flinders University Palaeontology Laboratory using a combination of acetic acid (5–10%) etching and mechanical methods, e.g., rock saws and pneumatic micro-jack tools^38^. For comparative specimens used, see Supplementary 2.

**Terminology.** Higher-level systematic nomenclature follows Aplin and Archer^86^, with the exception of: the use of the suborder Phalangeriformes Szalay^87^, sensu Woodburne^88^; the superfamily Vombatoidea for the clade that includes Vombatidae + Mukupirnidae + Maradidae following Beck et al.^30^ and Crichton et al.^39^; and the subordinal placement of Thylacoleonidae as Diprotodontia incertae sedis following Beck et al.^5^. We also use ?Wynyardiidae to refer to species within the genera *Namilamadeta* and *Muramura*, because their inclusion within the family Wynyardiidae has never been robustly demonstrated^33,89–91^. Biostratigraphic nomenclature follows Woodburne et al.^28^, Archer et al.^92^ and Travouillon et al.^93^. The ages of vertebrate bearing localities from the Namba and Etadunna Formations follows Woodburne et al.^28^ and Megirian et al.^29^, and those of Riversleigh World Heritage Area follow Archer et al.^92^ and Travouillon et al.^93^ sensu Woodhead et al.^53^.

Molar position homology follows Luckett^94^. Molar cusp nomenclature follows Rich et al.^95^ with the exception of: the structure reported therein as the hypocone, which is here referred to as the metaconule following Tedford and Woodburne^96^; and the buccal-most cusps on the upper molars in diprotodontians, which are discussed in Supplementary 1.

**Institution abbreviations**. *AR* Archer Collection, University of New South Wales, Sydney, New South Wales; *AMNH* Department of Vertebrate Paleontology, American Museum of Natural History, New York, U.S.A.; *FUR* Palaeontology Department, College of Science and Engineering, Flinders University, Bedford Park, Adelaide, South Australia; *NMV P* Palaeontology, Museum Victoria, Melbourne; *NTM P* Museum of Central Australia, Museum and Art Gallery of the Northern Territory, Alice Springs; *QM F*, Queensland Museum Fossil Collection, Brisbane; S *SAMA P* Palaeontology, South Australian Museum, Adelaide, South Australia.

**Phylogenetic analyses.** To assess the phylogenetic relationships of *Lumakoala blackae*, we adopted the morphological dataset from Crichton et al.^39^, built on that of Beck et al.^30^, comprising 124 craniodental and 20 postcranial characters, which were scored for 46 taxa. We included a further 13 taxa, for a total of 59, namely: *Lumakoala blackae* gen. et sp. nov., *Koobor notabilis, Stelakoala riversleighensis* and *Invictokoala monticola* (Phascolarctidae); *Thylacotinga bartholomaii* (putative polydolopimorphian); *Djarthia murgonensis* (Australidelphia); *Naraboryctes philcreaseri* (Notoryctemorphia); ‘*Bulungu* spp.’ (Peramelemorphia); *Nimbacinus dicksoni*, *Muribacinus gadiyuli* and *Badjcinus turnbulli* (Thylacinidae); as well as *Keeuna woodburnei* and *Ankotarinja tirarensis* (putative dasyuromorphians). To accommodate additional outgroup agreodontians, and changes to the homology of stylar cusp B in Diprotodontia, while minimizing pseudoreplication: three characters were deleted (char. 27, Lophodonty; 37, Preparacrista; 49, Relative placement of stC on M1); 19 were modified; and 17 were added. The resulting character matrix comprised 138 craniodental and 20 post cranial characters (see Supplementary 5 & 6).

Undated Bayesian analysis of the morphological dataset was carried out in MrBayes 3.2.7a ^97^, using the Markov Chain Monte Carlo (MCMC) approach, with gamma rate variability implemented for morphological data maintaining the assumption that only variable characters were scored. The Bayesian analyses were run for 15 million generations, using four independent runs of four chains (one cold and three heated chains, with the temperature of the heated chains set to the default value of 0.2), sampling trees every 1000 generations and a burn-in fraction of 25%. The post-burn-in trees were summarised using a majority rule consensus of all compatible groups, with Bayesian posterior probabilities as support values.

Maximum parsimony analyses were also performed on the morphological dataset, in TNT version 1.5 ^98^. The tree search involved an initial “new technology” search with sectorial search, ratchet, drift, and tree fusing that was run until the same minimum tree length was found 1000 times. From these saved trees a “traditional” search was applied using the tree bisection resection (TBR) swapping algorithm, with the resulting most parsimonious trees combined into a strict consensus tree. Support values for branch nodes were calculated using 2000 standard bootstrap replicates, implemented using a “traditional” search, which results in output as absolute frequencies.

Additional parsimony analyses were implemented in PAUP 4.0a169^99^, to test whether there were significant differences in the tree length and character state distribution of an alternative topological hypothesis, which included a positive constraint enforcing *Lumakoala blackae* within Phascolarctidae. To statistically compare these phylogenetic hypotheses, a single most parsimonious tree was randomly chosen from the unconstrained analyses, and compared against 50 random equal-most parsimonious trees from the constrained analyses using a one-sided Templeton Test^100^. This differs from a pairwise Templeton test in that the P value is halved^101^.

### Supplementary Information

Supplementary Information.

## Data Availability

Data generated and analysed during this study are included in this published article and its supplementary information file (available at https://doi.orgTBA).
