# Supplementary material for: A probable koala from the Oligocene of central Australia provides insights into early diprotodontian evolution
Source: Sci Rep. 2023 Sep 4;13:14521. doi: 10.1038/s41598-023-41471-0 (PMC10477348; doi:10.1038/s41598-023-41471-0)
Supplement: Supplementary file 1 — Supplementary Information. [file 41598_2023_41471_MOESM1_ESM.docx]

SUPPLEMENTARY INFORMATION FOR

**A probable koala from the Oligocene of central Australia provides insights into early diprotodontian evolution**

**Arthur I. Crichton^*^, Robin M. D. Beck, Aidan M. C. Couzens, Trevor H. Worthy, Aaron B. Camens, and Gavin J. Prideaux**

**This document includes:**

**Part 1. Stylar cusp nomenclature and homologies**

**Part 2. Measurements and comparative specimens**

**Part 3. *Madakoala* sp. cf. *M.* *devisi* description and comparisons**

**Part 4. *Nimiokoala* sp. indet. description and comparisons**

**Part 5. List of morphological characters**

**Part 6. Morphological matrix in NEXUS format**

**Part 7. Trees from phylogenetic analyses**

**Part 8. *Lumakoala blackae* body mass estimates**

**Part 9. Similarities between the Tingamarran taxa and *Lumakoala blackae***

**References**

**Part 1. Stylar cusp nomenclature and homologies**

The earliest definitive metatherians (e.g., *Kokopellia*, early deltatheroidans) are characterized by a well-developed stylar cusp that is connected to the paracone by the preparacrista, and which is referred to as the stylocone^1-3^. Another stylar cusp (the parastyle) may be present anterior to the stylocone, at or close to the anterobuccal corner of the tooth, but there are no well-developed stylar cusps present more posteriorly, although poorly defined cuspules may sometimes be present. Later tribosphenic metatherians, however, typically have additional, well-developed stylar cusps posterior to the stylocone. Collectively, these stylar cusps are conventionally referred to as A–E, following Clemens^4^, based on their position on the stylar shelf relative to the paracone and metacone, as follows: stylar cusp A (or parastyle), anterobuccal to the paracone; stylar cusp B (or stylocone), buccal to the paracone, and typically connected to the latter cusp by the preparacrista; stylar cusp C (or mesostyle), posterobuccal to the paracone and anterobuccal to the metacone, and at, or close to, the ectoflexus (if present); stylar cusp D, buccal to the metacone; stylar cusp E (or metastyle), posterobuccal to the metacone. Two stylar cusps may sometimes be present in approximately the same position, for example, there are two cusps in the B position in specimens of the Late Cretaceous *Aquiladelphis*^5^, and “twinned” cusps in the C position have been reported in a range of metatherians.

However, strict application of the purely position-based nomenclature of Clemens^4^ for stylar cusps fails to take into account likely homologies, a point specifically acknowledged by Clemens^4^ himself. For example, in dasyuromorphians, the large stylar cusp present in the posterior half of the stylar shelf (level with or posterior to the ectoflexus) may correspond topographically to the C, D, or E positions, but appears to be homologous in each case. In their survey of marsupials as a whole, Beck et al.^6^ discussed this issue, and followed a scheme in which the large cusp in the anterior half of the stylar shelf was consistently identified as stylar cusp B (with this cusp often, but not always connected to the preparacrista), and the large cusp in the posterior half of the stylar shelf was consistently identified as stylar cusp D.

In the new phascolarctid described here, use of the strictly positional scheme of Clemens^4^ would mean that the two large cusps in the centre of the stylar shelf, both of which are posterobuccal to the paracone and anterobuccal to the metacone, would probably be identified as twinned StC. Alternatively, the anterior of these two cusps (which lacks a connection to the preparacrista) could be referred to as StC, and the posterior cusp could be referred to as StD. However, species in the ?wynyardiid genus *Muramura* show two prominent stylar cusps, one immediately anterior to the ectoflexus and one immediately posterior, that are plausibly homologous based on their overall morphology with those of *Lumakoala blackae*, and yet the anterior of these is connected to the preparacrista (see Pledge^7^: fig. 19.2). Thus, the presence or absence of a connection to the preparacrista does not appear to be a robust criterion for determining whether a stylar cusp as StB (see Clemens^4^: 3; Beck et al.^8^: 756). Under the scheme of Beck et al.^6^, the two prominent stylar cusps in *L. blackae* would be identified as StB and StD, and hence sharing at least primary homology (sensu de Pinna^9^) with the two enlarged stylar cusps seen in many peramelemorphians, paucituberculatans, polydolopimorphians, and several other bunodont metatherian taxa.

**Part 2. Measurements and comparative specimens**

Measurements were made using Mitutoyo digital calipers (model No CD-8”C), rounded to 0.01 mm (Fig. S1; Table S1–2). Morphological comparisons were made directly from the specimens or casts thereof using an Olympus SZX12 microscope. The following exceptions drew upon published descriptions and figures^10-19^: *Priscakoala lucyturnbullae*; *Nimiokoala greystanesi*; *Litokoala kutjamarpensis*; *Litokoala kutjamarpensis*; *Litokoala dicksmithi*; *Litokoala garyjohnstoni*; *Koobor notabilis*; *Invictokoala monticola*; *Stelakoala riversleighensis*; *Chulpasia jimthorselli*; and *Thylacotinga bartholomaii*.


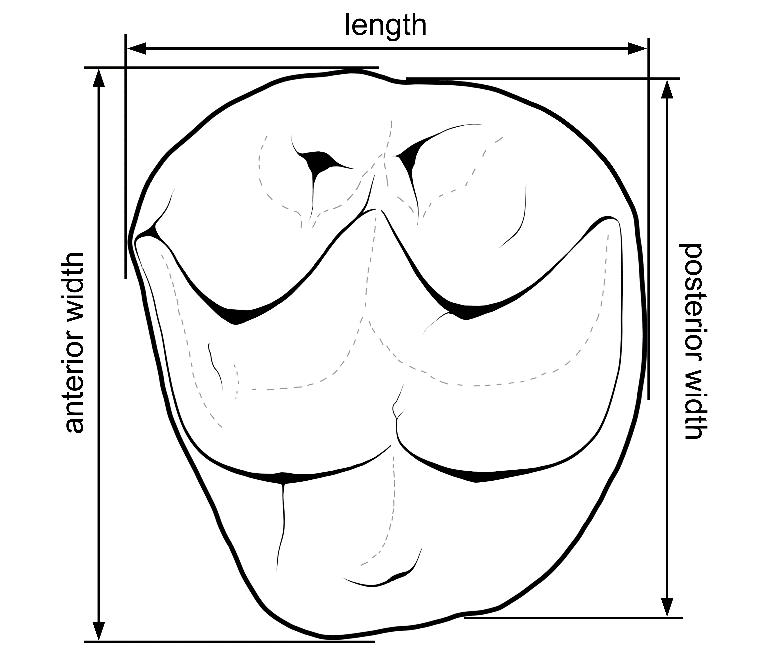


**Figure S1**. Diagram of *Lumakoala blackae*, gen. et sp. nov., LM?2 or 3 (Holotype, NTM P12012) from occlusal view showing orientation of measurements.

**Table S1**. Measurements (in mm) of upper dentition from select phascolarctid taxa. Measurements were made in this study for: *Madakoala* sp. cf. *M. devisi*, *Madakoala devisi*, *Lumakoala blackae* and *Litokoala ‘thurmerae’* (considered a nomen dubium^19^); and were taken from Black et al.^12^ for *Priscakoala lucyturnbullae*, Louys et al.^11^ for *Litokoala garyjohnstoni* and *L. kutjamarpensis*, Black et al.^19^ for *L.* dicksmithi, and Black et al.^14^ for *Nimiokoala greystanesi*. Symbols: >, greater than; *, approximately.

| Specimen | M1 | | | M2 | | | M3 | | | M4 | | |
| --- | --- | --- | --- | --- | --- | --- | --- | --- | --- | --- | --- | --- |
|  | L | AW | PW | L | AW | PW | L | AW | PW | L | AW | PW |
| *Lumakoala blackae* | | | | | | | | | | | | |
| NTM P12012 |  |  |  | 4.79 | 5.38 | 5.04 |  |  |  |  |  |  |
| NTM P12013 |  |  |  | 5.00* | 5.40* | 5.03* |  |  |  |  |  |  |
| *Priscakoala lucyturnbullae* | | | | | | | | | | | | |
| QM F20203 | 6.68 | 6.24 | 7.14 | 6.82 | 6.86 | 7.10 | 6.39 | 6.10 | 5.42 |  |  |  |
| QM F54563 |  |  |  | 7.14 | 6.73 | 6.68 |  |  |  |  |  |  |
| *Litokoala garyjohnstoni* | | | | | | | | | | | | |
| QM F51405 | 6.15 | 5.48 | 5.87 | 5.27 | 5.86 | 5.45 | 5.05 | 5.29 | 4.56 |  |  |  |
| QM F51406 |  |  |  |  |  |  |  |  |  | 4.02 | 4.20 | 3.44 |
| *Litokoala ‘thurmerae’* | | | | | | | | | | | | |
| SAMA P30159 |  |  |  |  |  |  | 4.47* | 4.51 | 3.90 |  |  |  |
| *Litokoala kutjamarpensis* | | | | | | | | | | | | |
| QM F51382 (left) | 5.85 | 5.08 | 5.03 | 5.46 | 5.38 | 5.01 | 5.37 | 5.03 | 4.37 | 4.69 | 4.13 | 3.10 |
| *Litokoala dicksmithi* | | | | | | | | | | | | |
| QM F54567 (left) | 6.64 | 5.73 | - | 5.53 | 5.48 | 4.79 | 5.51 | 5.18 | 4.23 | 4.43 | 3.79 | 2.58 |
| *Nimiokoala greystanesi* | | | | | | | | | | | | |
| QM F30482 (left) | 6.45 | 5.94 | 5.66 | 6.05 | 6.18 | 5.52 | 5.70 | 5.67 | 4.63 |  |  |  |
| *Madakoala* sp. cf. *M. Devisi* | | | | | | | | | | | | |
| NTM P14005 | 7.88 | 7.46 | 7.52 |  |  |  |  |  |  |  |  |  |
| *Madakoala devisi* | | | | | | | | | | | | |
| SAMA P58196 | 7.95 | >7.80 | 7.56 | 7.64 | >7.45 | >7.0 |  |  |  |  |  |  |
| SAMA P58195 | 7.70 | >7.30 | 7.10 | 7.50 | >7.45 | 7.0 | 7.30 | >7.1 | 6.41 |  |  |  |
| AMNH 102416 | 7.9 | >7.35 | >7.33 | 7.60 | 7.90 | 7.53 | 7.68 | 7.41 | 6.7 | 6.20 | >5.3 | 4.9 |

**Table S2**. Measurements (in mm) of lower dentition from *Madakoala* sp*.* cf. *M. devisi*, *Madakoala devisi* and *Madakoala wellsi*. Measurements made in this study. Symbols: *, approximately.

| Specimen | m1 | | | m2 | | | m3 | | | m4 | | |
| --- | --- | --- | --- | --- | --- | --- | --- | --- | --- | --- | --- | --- |
|  | L | AW | PW | L | AW | PW | L | AW | PW | L | AW | PW |
| *Madakoala* sp. cf. *M. Devisi* | | | | | | | | | | | | |
| NTM P12018 | - | - | 5.20 | - | 5.11* | - | 7.50 | 4.94 | 4.75 | 7.36 | 4.37 | 4.14 |
| NTM P12017 |  |  |  |  |  |  | - | - | 4.76 |  |  |  |
| *Madakoala devisi* | | | | | | | | | | | | |
| AMNH 102242 | 7.76 | 4.74 | - | 7.61 | 4.95 | - | 7.47 | - | 4.75 | 7.40 | 4.41 | 4.12 |
| *Madakoala wellsi* | | | | | | | | | | | | |
| SAMA P24793 | 8.39 | 5.22 | 5.97 | 8.11 | 5.81 | 6.07 | 7.77 | 6.65 | 5.82 | 7.86 | 5.12 | 4.81 |

**Part 3. *Madakoala* sp. cf. *M.* *devisi***

Material. NTM P14005, an M1; NTM P12017, a right paracone (M2 or M3); NTM P12015, a left m3 talonid; NTM P12016, a partial right lower molar row preserving m1 hypolophid, crushed m2 and complete m3-4.

**Lower dentition.** The size and morphology of the lower molars (NTM P12015 and NTM P12016) compares well with species of *Madakoala*, having: moderate crown height; a simple very weakly crenulated enamel surface, and a cristid that descends the buccal face from near the anterior terminus of the cristid obliqua on m1 (Fig. 4a–c). The lower molars differ from those of species of *Perikoala* in: being larger; relatively narrower; markedly less crenulated; and lacking a lingual shelf basal to the metaconid and entoconid. Among species of *Madakoala*, the molars are most similar in size (Table S2) and general morphology to those of *M. devisi*; differing from *M. wellsi* in: having fewer and less prominent crenulations; being markedly narrower with greater medial constriction; and lacking a cristid descending the posterobuccal face of the protocone on m2 (or protostylid ridge) (Fig. 4a–c). We limit referral of these specimens to *Madakoala* sp. cf. *M. devisi*, because NTM P12016 is missing the taxonomically informative m1 trigonid and the p3.

**Upper dentition.** The molar morphology (NTM P14005 and NTM P12017: Fig. 4d–g) is quite similar to that of *M. devisi* and species of *Perikoala*. The upper dentition of *M. wellsi* is not definitively known. In common with *M. devisi* and species of *Perikoala*: the terminus of the preprotocrista is not hypertrophied and consequently does not form a parastyle, and remnants of a continuous centrocrista (postparacrista+premetacrista) cross the transverse valley slightly lingual to stylar cusps B and D (Fig. 4f–g). The M1 is similar in size to that of *M. devisi*, and larger than that of *Perikoala* (15% greater length). The tooth is relatively low crowned, being comparable to those of species of *Perikoala*, but also falling within the range of known M1 specimens of *M. devisi*. The specimens exhibit abundant crenulations on the enamel surface, radiating down from the primary cusps, with prominent ridges also on the buccal basins of the paracone and metacone. The molars of species of *Perikoala* exhibit very similar radiating crenulations to those on NTM P14005, which are comparatively more abundant and prominent than those in *M. devisi*. Notwithstanding: enamel surface ornamentation on upper molars of *M. devisi* is intraspecifically variable; the upper molars generally bear more strongly crenulated surfaces than the lower molars; and crenulation prominence often grades from anterior to posterior along the molar row. The M1 Differs from that of *M. devisi* and species of *Perikoala* in having greater separation between stylar cusps B and D, and less sharply angulate corners.

The right upper molar fragment (NTM P12017: L 2.92 mm; W 3.14 mm) preserves a paracone and paraconule (Fig. 4d–e). It exhibits generally similar morphology to that of NTM P14005, with subtle differences that likely relate to their differing molar positions, wherein the former is thought to derive from an M2 or M3 and the latter is an M1. The paracone buccal basin of NTM P12017 is smaller than that on M2 or M3 in *M. devisi* by approximately 20%.

**Part 4. *Nimiokoala* sp. indet.**

**Material**. NTM P12018, a left protocone (tooth position unknown); and NTM P12019, a right protoconid (tooth position unknown).

**Description**. Two isolated phascolarctid molar fragments compare best with species of *Nimiokoala* (Fig. 4h–k). The specimens share with one another moderate crown height, high enamel surface complexity, and size roughly intermediate between *L. blackae* and *Madakoala* sp. cf. *M. devisi*. Notwithstanding, the structures preserved on NTM P12019 are noticeably larger relative to those on P12018 (Fig4. H–K). The left upper molar fragment (NTM P12018) preserves a damaged protocone (Fig. 4h–i). The specimen measures 2.73 mm in length and 2.87 mm in width. The buccal face of the protocone is vertical, being far steeper than the lingual face. The postprotocrista is strongly curvilinear, descending steeply posteriorly, and then forming a right angle as it curves and continues posterobuccally. There is no indication of a lingual cingulum. A small paraconule is positioned posterobuccal relative to the apex of the protocone. Anterior to the paraconule is missing due to damage. It is possible that the paraconule was partitioned (as in *N. greystanesi*), and all that remains is the posterior component. By comparison, in species of *Litokoala*, the paraconule is structurally uniform, and positioned almost entirely anterior relative to the apex of the protocone. In common with *N. greystanesi*, the paraconule is connected to a prominent crista that rises anterobuccally to the apex of the protocone.

A right lower molar fragment NTM P12019 (L 4.10 mm; W 3.00 mm) preserves a protoconid and a prominent neomorphic cuspid lingual to it in the longitudinal valley (Fig. 4j–k). The presence of a prominent cuspid between the protoconid and metaconid is known only from species of *Nimiokoala*. From the anterior extremity of the cristid descending anteriorly from the neomorphic cuspid, the remnant of a cristid ascends lingually, presumably towards the apex of the metaconid. From the posterior extremity of the cristid descending posteriorly from the neomorphic cuspid, a subsidiary cristid ascends posteriorly to meet the postprotocristid. In common with species of *Litokoala* and *Nimiokoala* (to the exclusion of species of *Madakoala* and *Perikoala*): the postprotocristid is strongly crescentic rather than linear; and is buccally oriented at its terminus, rather than posterobuccally oriented. It differs from species of *Nimiokoala* in that the enamel surface is comparatively simple, with no structures that descend the lingual face of the protoconid, with exception of a weak central rib from the apex.

**Part 5. List of morphological characters**

New or modified characters are indicated by *

1. Number of upper incisors (ordered) (Beck et al.^20^: char. 1): (**0**) Five; (**1**) Four; (**2**) Three; (**3**) One.
2. First upper incisor (Beck et al.^20^: char. 2): (**0**) Not greatly broadened mediolaterally; (**1**) Greatly broadened and "strap-like". We opt to score *Vombatus ursinus* as 0&1 because, in some specimens, the I1 has markedly greater mediolateral width than labiolingual width.
3. Size of I1 relative to I2 (Black et al.^12^: char 2): (**0**) Equal or similar in length; (**1**) I1 length greater than that of I2.
4. Hypselodont i1/I1^21^: (**0**) Absent; (**1**) Present.
5. I2 alveolus shape from ventral view^21^: (**0**) anteroposteriorly narrow, oblong; (**1**) Subcircular.
6. Diastema between I3 and C1^21^: (**0**) Present; (**1**) Absent or very short.
7. Upper canine (Beck et al.^20^: char. 3): Present (**0**); Absent (**1**).
8. P1 (Beck et al.^20^: char. 4): Present (**0**); Absent (**1**).
9. P2 (Beck et al.^20^: char. 5): Present (**0**); Absent (**1**).
10. Bladelike P3 - ratio of maximum length to maximum width (Beck et al.^20^: char. 7): <1.5 (**0**); >1.5 (**1**).
11. Bladelike P3 (Beck et al.^20^: char. 8): Ridged (**0**); Smooth-sided (**1**).
12. Enamel extending down the buccal surface of P3/p3 and onto the root (ordered: modified from Beck et al.^20^: char. 11): (**0**) Absent; (**1**) Enamel extends onto root and terminates before the alveolus; (**2**) Enamel extends onto root and terminates roughly at the alveolus; (**3**) Enamel extends down root into alveolus.
13. Cusp number on primary anteroposterior crest of P3 (ordered)^21^: (**0**) Monocuspid; (**1**) Bicuspid; (**2**) Tricuspid; (**3**) More than three cusps.
14. Cusp number lingual to primary anteroposterior crest on P3 (ordered)^21^: (**0**) Zero; (**1**) One; (**2**) Two.
15. Posterobuccal cusp on P3 (Beck et al. ^20^: char. 9): Absent (**0**); Present (**1**).
16. Densely crenulated posterobuccal face of P3^21^: (**0**) Absent; (**1**) Present.
17. *Alignment of P3 relative to M1(ordered)^21^: (**0**) Aligned with buccal cusps on M1; (**1**) Aligned centrally; (**2**) Aligned with lingual cusps on M1.
18. *Length of P3 relative to M1 (ordered) (modified^21^, from^12,22,23^): (**0**) >120%; (**1**) 100–119%; (**2**) 80–99%; (**3**) < 79%.
19. Upper molar roots on lingual face in adults^21^; (**0**) Do not extend far out of alveolus; (**1**) Extend out of alveolus by a height ≥ crown height.
20. Enamel noticeably thicker on trailing edge (lingual surface on upper molars and buccal surface on lower molars) relative to leading edge of molars (buccal surface on upper molars and lingual surface on lower molars)^22^: (**0**) Absent; (**1**) Present.
21. Enamel tracts extending down lingual surface of upper molars and buccal surface of lower molars (Beck et al.^20^: char. 12): (**0**) Absent; (**1**) Present.
22. Hypselodont cheek teeth (Beck et al.^20^: char. 13): (**0**) Absent; (**1**) Present.
23. Molar shape (Beck et al.^20^: char. 14): (**0**) Not strongly bilobed; (**1**) Strongly bilobed.

1. Fully lophodont molars (Beck et al.^20^: char. 18): (**0**) Without midlink; (**0**) With midlink.
2. Anteriorly concave lower / anteriorly convex upper molar lophs (Beck et al.^20^: char. 19): (**0**) Absent; (**1**) Present.
3. Cristae on posterolingual face of paracone (Beck et al.^20^: char. 25): (**0**) Absent/weak; (**1**) Strongly developed. In most phascolarctids, a crista descends from the apex of the paracone down the posterobuccal face.
4. Protostyle (Beck et al.^20^: char. 23): (**0**) Absent; (**1**) Present.
5. Postprotocrista and postmetaconulecrista^21^: (**0**) Absent or poorly defined, with postprotocrista terminating before transverse valley and postmetaconulecrista terminating before meeting posterior cingulum; (**1**) Both well-defined, with postprotocrista meeting transverse valley and postmetaconulecrista continuing around posterior margin, emulating a posterior cingulum, respectively.
6. *Cusp (attributable to stC) between stB and stD: (**0**) Present; (**1**) Absent.
7. Outline of M1 (ordered)^21^: (**0**) Anterior moiety wider than posterior moiety; (**1**) Anterior moiety subequal in width to posterior moiety; (**2**) Anterior moiety narrower than posterior moiety.
8. *Size of metacone relative to paracone on M1 (ordered): (**0**) Larger; (**1**) Subequal; (**2**) Smaller.
9. *Relative transverse placement of metacone on M1 (ordered) (Modified from^21^): (**0**) Positioned buccally, closer to stylar cusp D than to metaconule; (**1**) Positioned roughly mid-width between stylar cusp D and metaconule; (**2**) Positioned lingually, closer to metaconule than stylar cusp D.
10. Preprotocrista and premetaconulecrista on M1^21^: (**0**) Absent; (**1**) Present.
11. Metaconule on M1 (modified^21^, from Beck et al.^20^: char. 20): (**0**) Absent or weakly developed – triangular molars; (**1**) Enlarged, forming posterolingual cusp – square molars.
12. Cuspate lingual cingulum / neomorphic cuspule on the anterolingual base of the metaconule of M1 (modified^21^, from Beck et al.^20^, char. 24): (**0**) Absent; (**1**) Present.
13. Crest descending lingually from paracone on M1 (protoloph)^21^: (**0**) Meets preprotocrista; (**1**) Meets protocone.
14. *Buccal end of preprotocrista is flared anteriorly/hypertrophied on M1, forming ‘parastyle’ (ordered) (modified from^21^): (**0**) Absent; (**1**) Present, forming small cusp or raised shelf; (**2**) Present, forming large cusp.
15. *Paracone linked to stB on M1 (ordered) (modified from^21^: (**0**) Paracone linked to stB anteriorly via preparacrista/anteriorly displaced lingual-stB-crista; (**1**) Paracone linked to stB posterobuccally via postparacrista/posteriorly displaced lingual-stB-crista; (**2**) stB is reduced to a prominent crista descending posterobuccally / posteriorly from paracone; (**3**) Crista descending posterobuccally / posteriorly from paracone weak/absent.

lingual-stB-crista refers to the neomorphic crest that descends lingually from the apex of stylar cusp B. In some vombatiforms, it is not definitively clear whether the crista that links between the paracone and stB is derived solely from the lingual-stB-crista, or whether this has merged with either the preparacrista (e.g. species of *Muramura*) or postparacrista (e.g. species of *Namilamadeta*, *Mukupirna*, vombatids, phascolarctids, and putatively diprotodontoids).

On M1 in *Raemeotherium* *yatkolai* (Pinpa LF: SAMA P43060, partial left and right maxillae), the paracone is linked posterobuccally to a cuspate structure in the stylar cusp B position, from which, a crista continues posteriorly to the bottom of the transverse valley. On M2–M4, a curvilinear crista descends posteriorly from the paracone to the bottom of the transverse valley, but with is no discernible cusp in the stylar cusp B position. This arrangement of structures in *R. yatkolai* is mirrored in *Namilamadeta* spp., *Mukupirna* spp., and basal vombatids, in accordance with a cascadal morphocline from anterior to posterior along the molar row. We interpret that on M1, in early diprotodontids (e.g., *Ngapakaldia tedfordi*, *Silvabestius* spp., *Nimbadon lavarackorum*) and palorchestids, the crista oriented posterobuccally from the paracone (often continuing posteriorly to the bottom of the transverse valley) represents the atrophied remnants of the lingual-stB-crista + postparacrista + stylar cusp B. With respect to palorchestids, this interpretation of stylar cusp B homology is consistent with Murray ^24,^and Black ^25^ (in which the structure is identified as stC). In most Neogene and Quaternary diprotodontids this structure is very weak or absent.

1. Clearly raised posterobuccal terminus of postmetacrista on M1 forming a cusp (‘stylar cusp E’)^21^: (**0**) Present; (**1**) Absent.
2. *Cusp at buccal margin between stD and posterobuccal terminus of postmetacrista, on M1 (attributable to stE): (**0**) Present; (**1**) Absent.
3. Postmetacrista on M1–M2 (ordered)^21^: (**0**) Absent; (**1**) Reduced, terminates before meeting buccal margin; (**2**) Present, meets buccal margin and becomes continuous with postmetaconulecrista/posterior cingulum.
4. *Paraconule on M1 and M2 (modified from Beck et al.^20^: char. 21): (**0**) Absent; (**1**) Present.
5. *Neometaconule on M1 and M2 (modified from Beck et al.^20^): (**0**) Absent; (**1**) Present.
6. On M1–M2, prominent cristae on buccal face link stB and stD^21^: (**0**) Absent; (**1**) Present.
7. *Metacone linked to stD on M1–M2 (ordered) (Modified from^21^): (**0**) Not linked; (**1**) Linked but cusps are still clearly distinct; (**2**) stD and metacone subsumed entirely into the metaloph.
8. *StB on M2: (**0**) Not linked to paracone by a crista; (**1**) Linked to paracone by a crista.
9. *Paracone linked to stB on M2 (ordered) modified from^21^: (**0**) Paracone linked to stB anteriorly via preparacrista/anteriorly displaced lingual-stB-crista; (**1**) Paracone linked to stB posterobuccally via postparacrista/posteriorly displaced lingual-stB-crista; (**2**) stB is reduced to a prominent crista descending posterobuccally / posteriorly from paracone; (**3**) Crista descending posterobuccally / posteriorly from paracone weak/absent.
10. *Postparacrista meets premetacrista on M2 (1) (modified from^21^): (**0**) Absent; (**1**) Present.
11. *Postparacrista meets premetacrista on M2 (2) (modified from^21^): (**0**) Near tooth mid-width; (**1**) Slightly lingual to stylar cusps.
12. *Angle formed by posterobuccal corner of M2 in occlusal view: (**0**) <60°; (**1**) >60°.
13. *Size of metacone relative to paracone on M2: (**0**) Markedly larger; (**1**) Slightly larger or subequal.
14. *In taxa for which stylar cusp B is not connected to the preparacrista on M2-M3, clearly raised anterobuccal terminus of preparacrista on M2-M3 forming a cusp (modified from^21^): (**0**) Absent; (**1**) Present.
15. *In taxa for which stylar cusp B is not connected to the preparacrista on M2-M3, height of stylar cusp B relative to anterobuccal terminus of preparacrista (ordered) (modified from^21^): (**0**) Taller; (**1**) Subequal in height, or very slightly shorter; (**2**) Noticeably shorter.
16. Posteriorly increasing (upper) molar gradient (ordered) (modified from Beck et al.^20^: char. 28): (**0**) M3 and M4 absent; (**1**) M4 absent; (**2**) M4 markedly smaller than M1: (**3**) Absent, M4 and M1 similar in size, or M4 slightly smaller; (**4**) Present, M1 clearly larger than M4.
17. M4 metaconule (Beck et al,^20^: char. 27): (**0**) Absent/ significantly reduced and retracted towards posterior cingulum; (**1**) Distinct, cuspate.
18. Number of lower incisors (ordered) (Beck et al.^20^: char. 29): (**0**) Four; (**1**) Three; (**2**) Two; (**3**) One.
19. Anterior lower incisor (Beck et al.^20^: char. 30): (**0**) Not greatly enlarged; (**1**) Greatly enlarged and procumbent.
20. Dorsal surface of procumbent incisor (Beck et al.^20^: char. 31): (**0**) Not strongly concave; (**1**) Strongly concave, shovel-shaped.
21. Crown on i1^21^: (**0**) Enamel of relatively uniform thickness encapsulates i1 crown; (**1**) Enamel on the lingual face is absent, or much thinner than that on the labial face.
22. *Inclination angle of i1 (ordered) (modified^21^, from^12,22,26-28^): (**0**) High (greater or equal to 30 degrees); (**1**) Low (less than 30 degrees); (**2**) Negative inclination (inclined ventrally).
23. Cusp number on primary anteroposterior crest of p3 (ordered)^21^: (**0**) Monocuspid; (1) Bicuspid; (**2**) Tricuspid; (**3**) More than three cusps.
24. *Cuspate lingual cingulid on lower molars: (**0**) Absent; (**1**) Present.
25. Cristid obliqua (Beck et al.^20^: char. 37): (**0**) Present, well-developed; (**1**) Absent or indistinct.
26. Buccal precingulid on lower molars^21^: (**0**) Absent; (**1**) Present. In *Raemeotherium* *yatkolai*, the cingulid that spans the anterior margin of the lower molars continues buccally beyond the anterolingual terminus of the paracristid. Therefore, it is interpreted that this anterior cingulid derives (at least in part) from the precinguid. As such, the precingulid is coded as present in diprotodontoid that have a cingulid which spans the anterior margin of the lower molars.
27. *Hypoconid transversely linked to entoconid (modified from^21^): (**0**) Absent; (**1**) Present.
28. *In taxa for which the hypoconid and entoconid are transversely linked, the cristid that descends lingually from the apex of the hypoconid (ordered) (modified from^21^); (**0**) Terminates slightly lingual to apex of hypoconid; (**1**) Terminates close to mid-width between the apices of the hypoconid and entoconid; (**2**) Cannot be distinguished from the cristid that descends buccally from the apex of the entoconid.
29. Central cuspid on trigonid, between protoconid and metaconid^21^: (**0**) Absent; (**1**) Present. In *Namilamadeta* spp., a small central cuspid is variably present on the trigonid. The unworn lower molars of *Muramura* spp. are not known, though on m4 of the *M. pinpensis* specimen SAMA P.43044, a small central cuspid is discernible. Species of *Namilamadeta* and *M. pinpensis* are coded as 0&1.
30. Central cuspid on talonid between hypoconid and entoconid^21^: (**0**) Absent; (**1**) Present.
31. Metaconid on m1 (Beck et al.^20^: char. 32): (**0**) Present; (**1**) Absent.
32. Protostylid on m1 (Beck et al.^20^: char. 34): (**0**) Absent; (**1**) Present, cusp-like; (**2**) Present, vertically directed crest.
33. *Metastylid on m1, formed by posterior terminus of postmetacristid: (**0**) Absent; (**1**) Present.
34. Placement of protoconid relative to metaconid on m1^21^: (**0**) Protoconid positioned more anteriorly than metaconid; (**1**) Protoconid directly buccal to metaconid.
35. Width between the apices of the protoconid and metaconid, relative to that between the hypoconid and entoconid, on m1^21^: (**0**) Similar width; (**1**) Markedly narrower width between protoconid and metaconid.

1. Paracristid on m1^21^: (**0**) Prominent (meets anterior margin); (**1**) Weak (faint ridge that does not meet anterior margin).
2. Termination of paracristid on m1^21^: (**0**) Terminates in lingual position, anterior to metaconid; (**1**) Terminates in more buccal position, anterobuccal to metaconid.
3. *Paired ribs descending lingual face of entoconid from apex, on m1: (**0**) Absent; (**1**) Present.
4. Posterior face of hypoconid on m1–m3^21^: (**0**) Postcingulid absent; (**1**) Postcingulid present.
5. Postprotocristid on m2 and m3^21^: (**0**) Absent; (**1**) Present.
6. *Metastylid on m2–m3, formed by posterior terminus of postmetacristid (ordered) (modified from Beck et al.^20^: char. 36): (**0**) Absent; (**1**) Present, formed by only postmetacristid terminus; (**2**) Present, formed by termini of postmetacristid and preentocristid (metastylid fold of Black et al.^14^).
7. Apex of metaconid is anterior relative to apex of protoconid on m2-3 (Beck et al.^20^: char. 33): (**0**) No; (**1**) Yes.
8. Distinct and separate paraconid on m2–3 (Beck et al.^20^: char. 38): (**0**) No; (**1**) Yes.
9. Termination of m2–3 paracristid (Beck et al.^20^: char. 39): (**0**) Terminates in lingual position, anterior to metaconid; (**1**) Terminates in more buccal position, anterobuccal to metaconid.
10. Relative orientation of the postprotocristid + cristid obliqua on m2-m3^21^: (**0**) Anteroposteriorly straight; (**1**) Descend lingually.
11. *Relative placement of postprotocristid-cristid obliqua juncture on m2-m3 (ordered): (**0**) Buccal half; (**1**) Centrally; (**2**) Lingual half.
12. *Entoconid height as a percentage of metaconid height, on m3 (ordered): (**0**) >81%; (**1**) 31-80; (**2**) <30%.
13. *Hypoconulid on m3 (ordered): (**0**) Taller or subequal in height to entoconid (**1**) Markedly shorter than entoconid; (**2**) Absent.
14. *In taxa for which the hypoconulid is present, hypoconulid notch on m3: (**0**) Weak, or absent; (**1**) Deep.
15. *Premetacrista length relative to postmetacrista length, on M2 and M3 (ordered): (**0**) Much longer; (**1**) Subequal; (**2**) Much shorter.
16. *Trigonid markedly taller than talonid on m2-m4: (**0**) Present; (**1**) Absent.
17. *In taxa for which the trigonid is markedly taller than talonid, height of the protoconid relative to metaconid on m2-m4: (**0**) Subequal to or slightly less; (**1**) Markedly greater.
18. *Length between i1 and p3 (modified from^12,22,28^): (**0**) ≤ 30% cheek tooth row (p3-m4) length; (**1**) > 30% cheek tooth row (p3-m4) length.
19. Palate width at first molar^22^: (**0**) Wide, ≥ M1 width; (**1**) Narrow, < M1 width.
20. Placement of Infraorbital foramen relative to cheek teeth (ordered)^21^: (**0**) Anterior to P3; (**1**) Dorsal to P3; (**2**) Dorsal to M1; (**3**) dorsal to M2.
21. Intradiastemal ridges strongly coalesce: (**0**); Absent; (**1**) Present.
22. Incisive foramen in deep posteriorly-oriented V-shaped fossa that is formed independent from the intradiastemal ridges (modified from Beck et al.^20^: char. 41): (**0**) No; (**1**) Yes.
23. Incisive foramen in a deep ovate fossa (Beck et al.^20^: char. 42): (**0**) No; (**1**) Yes.
24. Incisive foramen located posteriorly (Beck et al.^20^: char. 43): (**0**) No; (**1**) Yes, the incisive foramina lie posteriorly, just anterior to P3, in a deep pocket at the rear of the diastema - the posterior position of the foramina correspondingly displaces the incisivomaxillary suture to a position just anterior to the cheek tooth row
25. Posterior palatal vacuities (maxillopalatine vacuities) (ordered: modified from Beck et al.^20^: char. 45): (**0**) Between maxilla and palatine; (**1**) Within palatine; (**2**) Absent.
26. Nasal aperture retracted beyond incisor arcade (ordered) (Beck et al.^20^: char. 46): (**0**) Absent or just posterior to incisor arcade; (1) Retracted to above diastema (or level of incisivomaxillary suture); (**2**) Retracted to above cheek tooth row.
27. Nasals (Beck et al.^20^: char. 47): (**0**) Not markedly broadened anteriorly; 1) Markedly broadened anteriorly.
28. Nasomaxillary suture length relative to nasopremaxillary suture length (dorsal view) (Beck et al. ^20^: char. 48): (**0**) Nearly same or greater; (**1**) Far less than naso-premaxillary suture length.
29. Direction of frontonasal suture from midline to lacrimal bone (dorsal view) (Beck et al.^20^: char. 49): (**0**) Lateral; (**1**) Rostrolateral.
30. Nasals contact lacrimals (Beck et al.^20^: char. 50): (**0**) No; (**1**) Yes.
31. Combined breadth of nasal bones in dorsal view (Beck et al.^20^: char. 51): (**0**) Much less than length; (**1**) Equal to or greater than length.
32. Postorbital/supraorbital process of frontal bone in dorsal view (ordered) (Beck et al.^20^: char. 52): (**0**) Small or absent; (**1**) Well-developed; (**2**) Laterally extensive, contacting frontal process of jugal to form complete postorbital bar.
33. Postorbital process (Beck et al.^20^: char. 53): (**0**) Formed by frontal only; (**0**) Formed by frontal and lacrimal.
34. Sagittal crest (Beck et al.^20^: char. 54): (**0**) Absent; (**1**) Present.
35. Infraorbital foramen (Beck et al.^20^: char. 55): (**0**) Round or oval; (**1**) Slit-like.
36. Masseteric process; ventral extent (ordered) (Beck et al.^20^: char. 56): (**0**) Absent or indistinct; (**0**) Short; (**1**) Elongate.
37. Masseteric process: lateral extent (Beck et al.^20^: char. 57): (**0**) Not markedly expanded laterally; (**1**) Greatly expanded laterally.
38. *Masseteric process composition (modified from Beck et al.^20^: char. 58): (**0**) Maxilla and jugal; (**1**) Jugal only; (**2**) Maxilla only.
39. Presence of a single and extensive area for attachment of masseter muscles extending from anterior to orbit to beneath orbit and traverses both maxillary and jugal bones (Beck et al.^20^: char. 59): (**0**) Absent or indistinct; (**1**) Forming prominent depression.
40. Lacrimal tubercle/tuberosity (Beck et al.^20^: char. 60): (**0**) Absent; (**1**) Present.
41. Palatine-lacrimal contact in orbit (Beck et al.^20^: char. 61): (**0**) Present; (**1**) Absent - frontal-maxilla contact.
42. Frontal-squamosal contact (Beck et al.^20^: char. 62): (**0**) Absent, alisphenoid-parietal contact; (**1**) Present.
43. Glenoid fossa 1 (Beck et al.^20^: char. 63): (**0**) Articular eminence planar or concave, mandibular fossa absent or indistinct; (**1**) Articular eminence planar, mandibular fossa well-developed; (**2**) Articular eminence strongly convex and broad
44. Postglenoid process (ordered) (Beck et al.^20^: char. 64): (**0**) Absent or weakly developed; (**1**) Present, vertical; (**2**) Present, ventral edge curving posteriorly to partially floor external auditory meatus.
45. Position of postglenoid foramen (Beck et al.^20^: char. 65): (**0**) Posterior to postglenoid process and bounded medially by petrosal; (**1**) Anteromedial to or in line with postglenoid process; (**2**) Posteromedial to postglenoid process within squamosal; (**3**) Within epitympanic fenestra, surrounded by bony septum.
46. Tympanic cavity roof elements (ordered) (Beck et al.^20^: char. 66): (**0**) Alisphenoid (no squamosal contribution); (**1**) Alisphenoid and squamosal; (**2**) Squamosal (no alisphenoid contribution).
47. Tympanic floor elements (ordered) (Beck et al.^20^: char. 67): (**0**) Alisphenoid; (**1**) Alisphenoid and squamosal; (**2**) Squamosal.
48. Tympanic wing, whether squamosal or alisphenoid (ordered) (Beck et al.^20^: char. 68): (**0**) Absent; (**1**) Does not contact exoccipital; (**2**) Contacts exoccipital.
49. Epitympanic fenestra developed in postglenoid cavity (Beck et al.^20^: char. 69): (**0**) Absent; (**1**) Present.
50. Non-auditory sinuses (Beck et al.^20^: char. 70): (**0**) Absent; (**1**) Present.
51. Posterior epitympanic sinus (Beck et al.^20^: char. 71): (**0**) Absent / weak; (**0**) Moderate to deep.
52. Interparietal (Beck et al.^20^: char. 72): (**0**) Present, large; (**1**) Absent, very small or polymorphic.
53. Ventrolaterally flared mastoid process on occiput (Beck et al.^20^: char. 73): (**0**) Absent; (**1**) Present.
54. Size of paroccipital processes (Beck et al.^20^: char. 74): (**0**) Small; (**0**) Large.
55. Palate (Louys et al.^29^: char. 100): (**0**) Not strongly arched; (**1**) Strongly arched.
56. Posterior extent of mandibular symphysis on mandible (ordered) (Beck et al.^20^: char. 75): (**0**) Anterior to p3; (**1**) Below p3; (**2**) Below m1; (**3**) Below m2-3.
57. Flared masseteric eminences on mandible (Beck et al.^20^: char. 76): (**0**) Absent/weak; (**1**) Moderately to strongly flared.
58. Masseteric foramen (Beck et al.^20^: char. 77): (**0**) Absent; (**1**) Present.
59. Fused mandibular symphysis (Beck et al.^20^: char. 78): (**0**) Absent; (**1**) Present.
60. Angle of the ascending ramus relative to the longitudinal ramus (modified^21^, from^12,22,28,30^): (**0**) < 70°; (**1**) > 70°).
61. Mandibular foramen located deeply within pterygoid fossa^21^: (**0**) Absent; (**1**) Present.
62. Depth of masseteric fossa (mandible) (modified^21^, from^22,31^: (**0**) Shallow depression; (**1**) Deep and well-excavated.
63. Anterior mental foramen (ordered)^21^: (**0**) Ventral or anteroventral to p3 by < ½ m1 length; (**0**) Anteroventral to p3 by ≥ ½ m1 length; (**1**) Anteroventral to p3 by ≥ m1 length.
64. Origin of ascending ramus (ordered)^21^: (**0**) Posterolateral to m4; (**1**) Lateral to m4; (**2**) Lateral to m3.
65. Subarcuate fossa (Beck et al.^20^: char. 79): (**0**) Deep and well-excavated; (**1**) Shallow depression.
66. Humerus, deltoid ridge (Beck et al.^20^: char. 80): (**0**) Has no lateral overhang, or a very minor one; (**1**) Has a large flaring overhang.
67. Cuneiform, position relative to scapholunar (Beck et al.^20^: char. 81): (**0**) Directly lateral to scapholunar; (**1**) More proximal than the scapholunar.
68. Cuneiform, ridge between ulnar and pisiform facets (Beck et al.^20^: char. 82): (**0**) Middle section sinks below the rim of the cuneiform; (**1**) High rising throughout, straight.
69. Scaphoid, distal surface (Beck et al.^20^: char. 83): (**0**) Single facet, smooth; (**1**) Separated into two distinct facets.
70. Scaphoid, distolateral process (Beck et al.^20^: char. 84): (**0**) Absent; (**1**) Present.
71. Unciform, hamate process (Beck et al.^20^: char. 85): (**0**) Large, hooked and curves medially; (**1)** Reduced, not as hooked and does not curve as far medially.
72. MC II, proximal end (Beck et al.^20^: char. 86): (**0**) Mediolaterally compressed; (**1**) Expanded and concave.
73. MC III, facet for MC IV (Beck et al.^20^: char. 87): (**0**) Proximal only to ligament pit (no dorsal component); (**1**) Dorsal to ligament pit (no proximal component); (**2**) Dorsal and proximal to ligament pit.
74. MC III, facet for MC IV (Beck et al.^20^: char. 88): (**0**) Facing laterally or only slightly distally slanted; (**1**) Strongly distally slanted or completely facing distally.
75. MC III, magnum facet (Beck et al.^20^: char. 89): (**0**) Grooved or concaved; (**1**) Convex or flat to slightly convex.
76. MC III, MC II facet (Beck et al.^20^: char. 90): (**0**) Separate, distinct, and lateral to the magnum facet; (**1**) Continuous with the magnum facet.
77. Proximal phalanges, manus or pes, distal ends (Beck et al.^20^: char. 91): (**0**) Not tapered; (**1**) Dorsoventrally tapered, so that the articulation for the medial phalanx faces ventrally.
78. Pelvis, ilium (Beck et al.^20^: char. 92): (**0**) Only slightly dorsoventrally flattened, or mediolaterally flattened; (**1**) Greatly dorsoventrally flattened.
79. Fibula, lateral notch on distal end (Beck et al.^20^: char. 93): (**0**) Clearly present; (**1**) Extremely slight or absent.
80. Fibula, femoral facet (Beck et al.^20^: char. 94): (**0**) Not present; 1) Present.
81. Astragalus, lateral tibial facet (Beck et al.^20^: char. 95): (**0**) Mostly convex; (**1**) Mostly concave.
82. Astragalus, head (Beck et al.^20^: char. 96): (**0**) Wider than long; (**0**) Narrow, mediolaterally compressed, or length and width are equal.
83. Cuboid (Beck et al.^20^: char. 97): (**0**) No contact; (**1**) Contacts astragalus.
84. Entocuneiform, MT I facet (Beck et al.^20^: char. 98): (**0**) Saddle-shaped (concavo-convex); (**1**) Convex.
85. Metatarsal I, entocuneiform facet (Beck et al.^20^: char. 99): (**0**) Mostly convex; (**0**) Strong concave component.

**Part 6. Morphological matrix in NEXUS format**

#NEXUS

Begin data;

Dimensions ntax=59 nchar=158;

Format datatype=standard symbols="012345" gap=-;

Matrix

Didelphis_marsupialis 00001-0000-000001300000--0011022100-0010200100-1000103000-0000010-0000011000000010--110201100-0000000100101000-0000012001000001000001002100???????????0?11?0??

Perameles_bougainville 00001-0000-001000300000--0011022100-0010200010-0-00103010-0100010-0000011000000010--111200102-0000000100000000-00010100010011000000000020000001102000000001000

Galadi_speciosus 00001-0000-001000300000--0011022100-0010200100-1100103010-??00010-0000011000000010--111200101-00000001000-1000-000001000100010000000100200????????????????????

Bulunga_spp. ??????????????????0?000--001(0 1)022100-001020010101100--30??????0010-0000011000000010--1112001????????????????????????????????????????????20?????????????????????

Naraboryctes_philcreaseri 00001-00011000000100000--0011021100-0-11200100-1100003?10-0010010-0000011000000010--20020110?-??????????????????????????????????000010020?????????????????????

Djarthia_murgonensis ??????0000-00000??0?000--0110022100-0011200001-1100--30?????00010-0000001000100010--1102011??-?????????????????????????????????????????2?0???????????????0????

Keeuna_woodburnei ??????????????????0?000--0110022100-00?120010101100--30??????0010-0000001000100010--1102011??-????????????????????????????????????????????????????????????????

Ankotarinja_tirarensis ??????????????????0?000--0110022100-00?120010101100--30??????0010-0000001000100010--1102011??-????????????????????????????????????????????????????????????????

Nimbacinus_dicksoni 100?1-00011001001100000--0111022100-001120011101000--3010-?000010-0000001000?00010--200201102-0000000100101000-00?101?001???00000000000200????????????????????

Muribacinus_gadiyuli ????????011001001100000--0111022100-001120011101000--3010-?000010-0000001000100010--200201103-0000?????????0????????????????????0??000020?????????????????????

Badjcinus_turnbulli 100?1-00??????????0?000--01110??100-001120011101000--30?????00010-0000001000100010--20020110?-??????????????????????????????????00?0???20?????????????????????

Ngapakaldia_tedfordi 2000100110-00100030000001--011--010102110--02120-1---4131001000112000001001010000----2--1-00100002101100001010101?1113211111?010001010021101110002000??1110101

Kolopsis_torus 20101-1110-02200020000001--011--010103110--02130-1---4131011001112000001001010000----2--1-101000020001000010200010111??(0 1)111??0102?0110001?0????1???????11?????

Neohelos_stirtoni 20101-1110-01200020000001--011--010103110--02130-1---4131011001112000001001010000----2--1-10100002000100001020001?1113211111?0102010100011????????????????????

Nimbadon_lavarackorum 20101-1110-01200020000001--011--010102110--02130-1---4131001001112000001001010000----2--1-10000002000100(0 1)0102000101113211111001020(0 1)(0 1)100111011101020001?01?????

Silvabestius_spp. 2010100110-01(1 2)00020000001--011--010102110--02120-1---313101?001112000001101010000----2--1-10100002001100101020001001132111110010??1???0???????????????????????

Litokoala_kutjamarpensis ???????1110021001200000--1111121111-211121111110-111?20?????200(0 1)0-01011010000121010202-11-00???000????????1?102????1111110010010?????????0????????????????????

Litokoala_dicksmithi 20?0100110-021001(2 3)00000--1111021110-211121111110-111?20????????????????????????????????1??0000000?00000010?0102010????????????????????????????????????????????

Madakoala_spp. 20101001100031100200000--1011121110-011121101111111112031000300(0 1)0-00011010000110010102-11-0000????????????????2????????????????02??0?0001?????????????????????

Nimiokoala_greystanesi 2010100110-021101300000--1111021110-211121111110-111?2031000200(0 1)(0 1)-11010010000101010202-11-0010000?00?1?0??101020110???1??0010??020100000?0????????????????????

Perikoala_robustus ???????110?03110??00000--0011121110-011121(0 1)0?111111112031????001(0 1)-00011?????0111010102-11-?0????????????????????????????????????2????0????????????????????????

Phascolarctos_cinereus 201010011100(2 3)1(0 1)01200000--111102111(0 1)-(1 2)11121(0 1)11110-11122131000200(0 1)0-01011110110120010202-11-002000010010011010102011(0 1)11110110100102111100100011100000??0001?????

Stelakoala_riversleighensis ?????????????????????????????????????????????????????????????0000-?10111100101????????????????????????????????????????????????????????????????????????????????

Priscakoala_lucyturnbullae ????1??????????0??00000--0111221110-0111200(0 1)111111110????????0010-???110100001????0????1??????????????????????????????????????????????????????????????????????

Lekaneleo_roskellyae 20101000011010000000000-0001100010000-1120000??1111002?2100010000-0010001010010-0?????-0??000-000000010010100--0000112111001000010100000?0????????????????????

Wakaleo_oldfieldi 20?0??0??1101000?000000-0001100010000-1120000??1111001-?100?10000-0012001010010-0?0012-01-00?-?????????????????????????????????01??00???-???????????????????-?

Wakaleo_vanderleuri 20101000111010000000000??001100010000-1120000??1111001-?100010000-0012001010010-0?0012-01-000-000000010010100--00?0112?110?1?01?1?100000-???????????????????-?

Sedophascolomys_medius 31?1--111??3???0??01111??????????1???????????????1???3?31??????????????????????????????????0?01011?0???????????????????????????131110??1??????????????????????

Phascolonus_gigas 3111--111??3???0?301111??????????1???????????????1???3?31011??????????????????????????????00?0101120????0?010--1???2022-0??1?1?1311101112?1111?11?1?1111110???

Ramsayia_magna 3011--111??3???0?301111??????????1???????????????1???3?31011??????????????????????????????1?10?0112001?1000?(0 1)101??????????1????121110?????????????????????????

Lasiorhinus_krefftii 30?1--111??3???0?301111??????????1?????????1????-1???3?31011??????????????????????????????111001011000(0 1)110000--110(0 1)202221001110031111?012?????????????????????

Lasiorhinus_latifrons 3011--1110-30000?301111-000????1?1010????001???0-1???3?31011000??????00????00?01110???????111001011000(0 1)110000--1101202221001110031111101211111011?11111111011?

Mukupirna_nambensis 2?1??10110-11?01211?000-000110101101011100011110-110021??????????1?????????????????????????0?00101?????????????0???00????????1????????????0??10?011?01?0??????

Vombatus_ursinus 3(0 1)11--111?131100?301111-000????0?10101???001?110-1?0?3?31011100?(0 1)100000?10?001011100??????11100001101100(0 1)0010--11012022210011100311111111111110111111111110111

Vombatus_hacketti 3(0 1)11--111??3???0?301111??????????1??????????????-1?????31011??????????????????????????????11?000011011?000010--11??202?(1 2)10?111?031111111??1???????????????????

Warendja_wakefieldi ?011???11??3???0?301111??????????1??????????????-1???3?31011??????????????????????????????10?0??0????1????100--0?1000222100?1??0211001?01?1???????????????????

Zygomaturus_trilobus 20101-1110-02200020000001--011--010103110--02130-1---4131010001112000001001010000----2--1-10100?02010??0??1020001??11??0101??01030011000110?11?10?0?01????????

Diprotodon_optatum 21111-1110-0(0 1)100030000001--011--010103110--02130-1---413101110111200000101-010000----2--1-10110002100100000020001?111??2??11?00020011000110111?10?0001?1??????

Euryzygoma_dunense 20111-1110-0(0 1)100030000001--011--010103110--02130-1---413101100111200000101-010000----2--1-1011000211??????102100???11??2??1??0?0??0110001?????????????????????

Pyramios_alcootense 20001-1110-00100020000001--011--010103010--02130-1---413100100111200000101-010000----2--1-1010???200???0???020?????????2???????02?011000??????????????????????

Pro._novaculacephalus 20??1-1110-00100030000011--011--010102110--02120-1---3131???000112000001001010000----2--1-102000?22011010010200011011?21(1 2)111?11021(0 1)110011?????????????????????

Palorchestes_painei 20?01-1110-00100030000011--011--010102110--02120-1---4131?02000112000001001010000----2--1-1030000220?101???0(1 2)0?0????1??2?1???11020?100011?????????????????????

Palorchestes_azael 20001-1110-0(0 1)100030000011--011--010102110--02120-1---4131102000112000001001010000----2--1-1030000220?1011100(1 2)0001112032??111?1103001100211????????????????????

Namilamadeta_spp 20110001110022000100000-0001111-1101011110001110-11002131011200111(0 1)00000101001010110-2--1-10100000100100(0 1)0102000011112211000?1102010000111????????????????????

Thylacoleo_carnifex 2010110001101000?000000--?0??0???0??0????000?????????0-(1 2 3)10001?0??-??12?????????-??????????00?-000000110020(0 1)00--0101123221111000000100000-(0 1)?11100001100?1??????

Nimbavombatus_boodjamullensis ?????10110-21(0 1)000301100-0??110???101?111???1??10-11??31??????????????????????1???1??02--1-?01???00?????????00--0?0????????????????????????????????????????????

Mukupirna_fortidentata 20100101100112010111000-000110101101011100011110-1100213101010011100000010000101?11002--1-0010010?00?1?0???0???0???????????????010?000002?????????????????????

Muramura_pinpensis 2010??01100022000100000-000111121101101110001100-11--2131010200110(0 1)0000010100101010102-11-10?0000000???000102000???112211000?11?10100001110????????????011????

Muramura_williamsi 20100001100022000100000-0001?1?2?101?01110001100-11--31310?1200110??000000100?01010102-11-101?000000???000102000???112211000?11010100?01110????????????011????

Thylacotinga_bartholomaii ????????????????????000--0(0 1)11??1100-0-11200100-111110?1?????00010-00??0??000000?10--11110?????????????????????????????????????????????????????????????????????

Rhizophascolonus_spp. ?011?????0-32(1 2)00?301100-000112101101011100011110-110??????1??1001100000010000101011002--1-??????????????????????????????????????????????0?????????????????????

Marada_arcanum ???0???????1???????1000-0?0??????????????00????????????3101111011100000010100101011002-?1-1?????????????????????????????????????0??000020?????????????????????

Raemeotherium_yatkolai ???0??0110-0?100020000001000?0--010?011100012120-1-0-3131011?001120000001010100001---2--1-0????????0??????????0?????????????????10?01?010?????????????????????

Koobor_notabilis ?????????0(0 1)011001200000--1011021110-111121011110-1112????????????-?????????????????????1???0????????????????1020??????????????????????????????????????????????

Invictokoala_monticola ??????????????????00000--0011???110???1?210?1110-1112????????????-?????????????????????1??????????????????????????????????????????????????????????????????????

Kuterintja_ngama ?????????0-02000?200000--0010221110-0-012000(0 1)110-111021310?1100110110001?0001100000202-11-1?????????????????20??????????????????2??01?010?????????????????????

Ilaria_spp. 2010000110-011101200000-0001112111(0 1)-010120001110-1110413100010011011000100001100000201111-00?001???????????02000???11?2(1 2)10???1?12101?00011??????111111????????

Lumakoala_blackae ???????????????????0000--0(0 1)112?111(0 1)-0?11210000-111110??????????????????????????????????1??????????????????????????????????????????????????????????????????????

Cercartetus_lepidus 2010100000-010000200000--001-10-11000-11200----1-11--202100110000-00000010100101010002-11-000-0000001100000000-0000011102001000010000000100??000????????1000??;

End;

**Part 7. Trees from phylogenetic analyses**


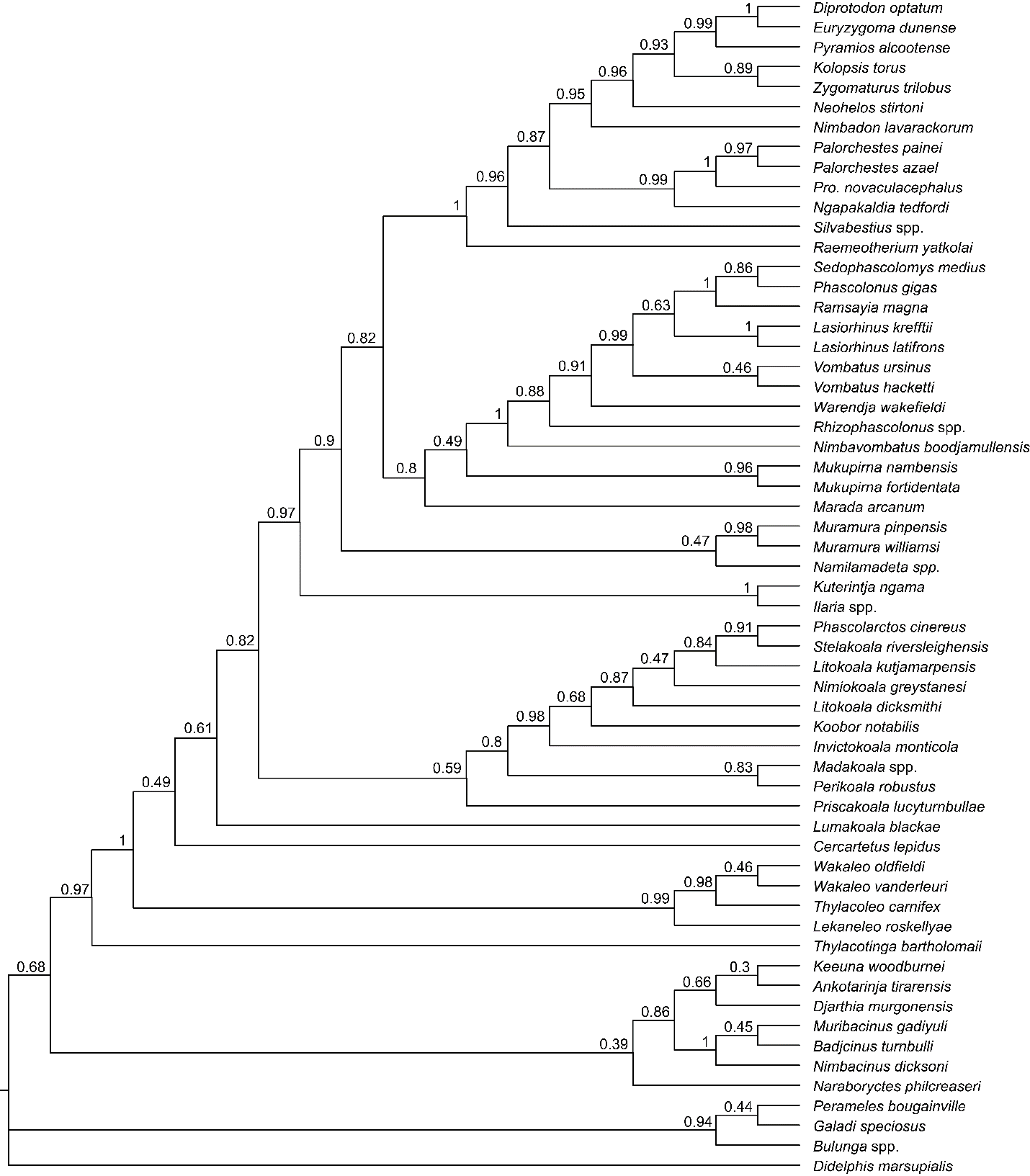


**Figure S2**. Phylogeny of vombatiforms that result from Bayesian analysis of the morphological dataset, without constraints. Presented as a majority rule consensus with numbers at nodes representing Bayesian posterior probabilities (BPP).

**
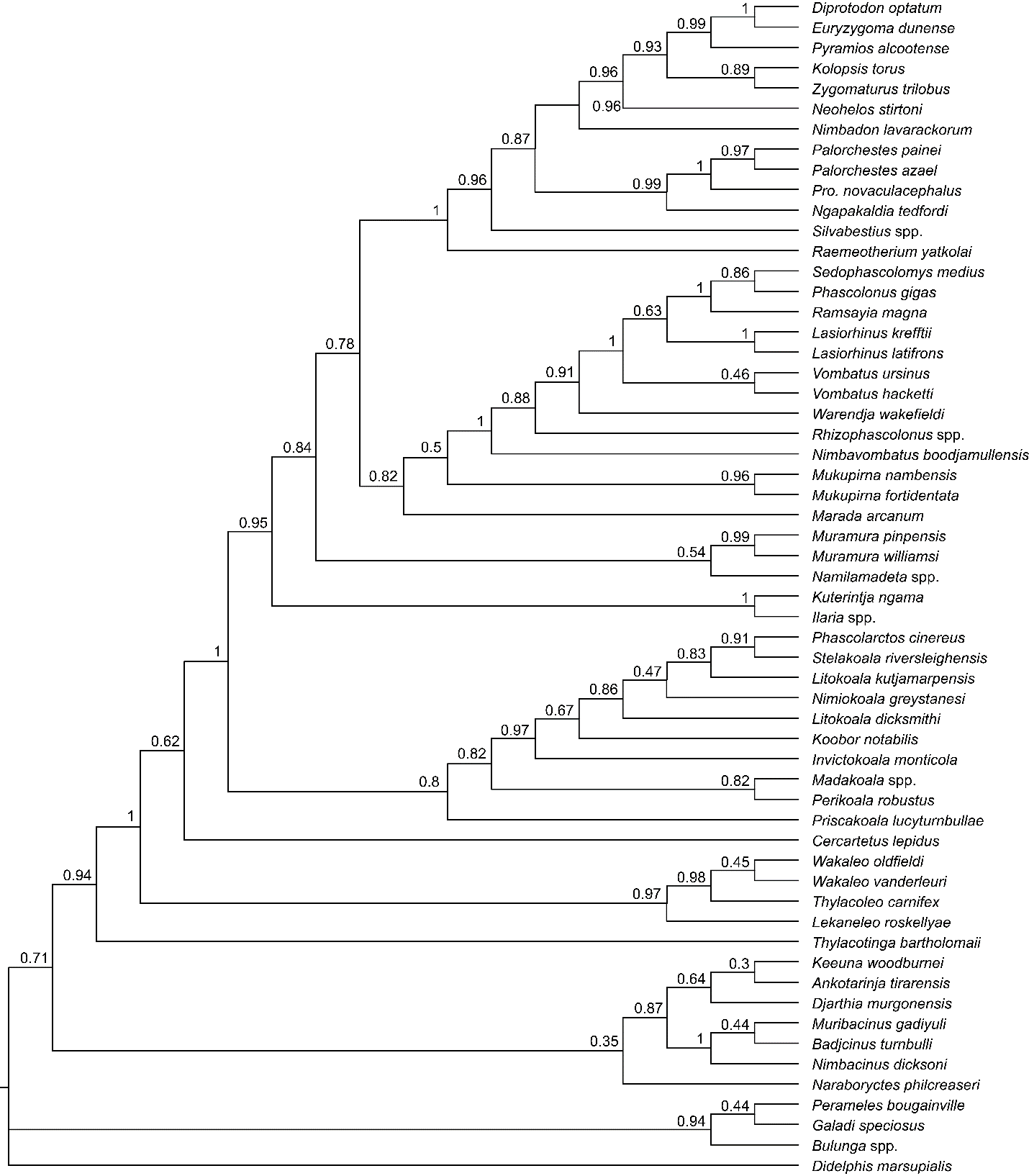
**

**Figure S3**. Phylogeny of vombatiforms, excluding *Lumakoala blackae*, that result from Bayesian analysis of the morphological dataset, without constraints. Presented as a majority rule consensus with numbers at nodes representing Bayesian posterior probabilities (BPP).

**
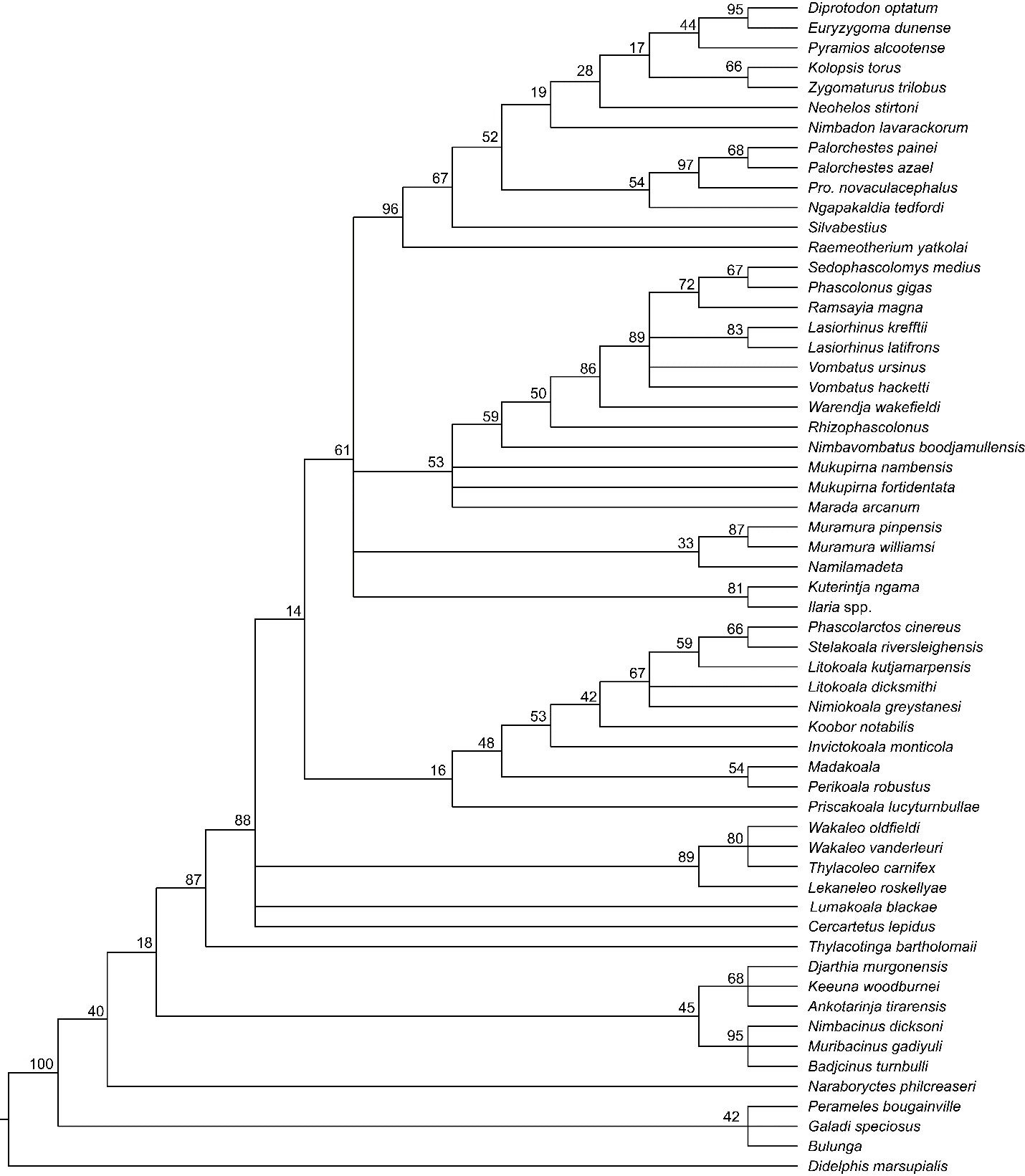
**

**Figure S4**. Phylogeny of vombatiforms that result from maximum parsimony analysis (strict consensus) of the morphological dataset without constraints. Numbers at nodes represent bootstrap support values.


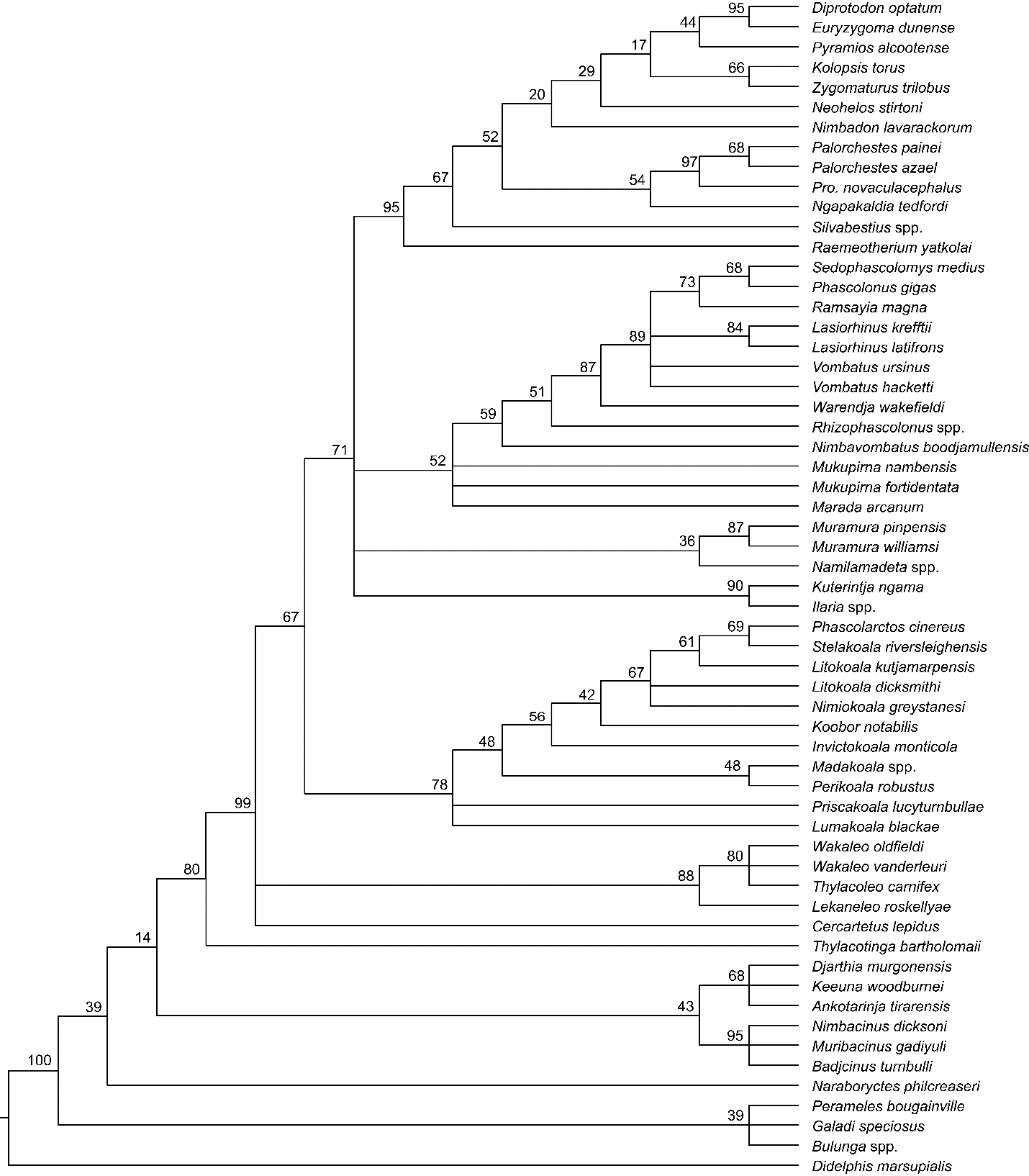


**Figure S5**. Phylogeny of vombatiforms that result from maximum parsimony analysis (strict consensus) of the morphological dataset, with a positive constraint forcing *Lumakoala blackae* within Phascolarctidae. Numbers at nodes represent bootstrap support values.

**Part 8. *Lumakoala blackae* body mass estimates**

**Table S3.** Body mass estimates (in kg) for *Lumakoala blackae*, based on measurements taken from upper molar specimens, presented for both M2 and M3 regression equations (of Myers^32^)

Abbreviations: *logBM*, log body mass; *M2L*, second upper molar length; *M3L*, third upper molar length.

| **Specimen** | **Variable x (mm)** | **Regression equation** | **Smearing estimate (%)** | **Body mass (kg)** |
| --- | --- | --- | --- | --- |
| NTM P12012 | M2L (4.79) | logBM=1.039+3.34 (log x) | 9.6 | 2.2 |
| NTM P12012 | M3L (4.79) | logBM=1.348+2.89 (log x) | 6.8 | 2.2 |
| NTM P12013 | M2L (5.0) | logBM=1.039+3.34 (log x) | 9.6 | 2.6 |
| NTM P12013 | M3L (5.0) | logBM=1.348+2.89 (log x) | 6.8 | 2.5 |

**Part 9. Similarities between the Tingamarran taxa and *Lumakoala blackae***

*Thylacotinga bartholomaii* and *Chulpasia jimthorselli* share a number of likely derived similarities with *Lumakoala blackae* that are absent in: basal members of Notoryctemorphia (e.g. *Naraboryctes philcreaseri,* and NTM P2815-6 in^33^); Peramelemorphia (e.g. *Yarala burchfieldi* and species of *Bulungu*); Dasyuromorphia (e.g. *Nimbacinus dicksoni*, *Mutpuracinus archibaldi*); and the dasyuromorph-like *Keeuna woodburnei* and *Ankotarinja tirarenensis*, which may be related to the possible stem-australidelphian *Djarthia murgonensis*^35,36^). These M2 attributes include: a paracone slightly smaller than metacone, rather than paracone markedly smaller than metacone; premetacrista subequal in length to postmetacrista, rather than postmetacrista noticeably longer; an angle formed by posterobuccal corner in occlusal view of >90°, rather than being <60°; protocone positioned posterolingual relative to paracone, rather than lingual or anterolingual (also in posterior molars of dasyuromorphians); retention of all structures on M4; stylar cusp B taller than stylar cusp D (also in some peramelemorphians and *Djarthia murgonensis*); and open buccal margin between the preparacrista terminus and stylar cusp B, as well as stylar cusp D and the postmetacrista terminus, respectively (also in notoryctemorphians). Symplesiomorphies retained by *T. bartholomaii*, *C. jimthorselli* and *L. blackae* include: a continuous postparacrista+premetacrista (= complete centrocrista); postparacrista and premetacrista that do not meet the stylar cusps B and D, respectively; preparacrista with a raised terminus forming stylar cusp A; stylar cusps B and D well-developed and somewhat conical. It is also worth pointing out that body size estimates for *L. blackae* sp. nov., of 2.2–2.6 kg, using length of the M2 specimens NTM P12012 and NTM P12013 following methods of Myers^32^, place it between those for *Thylacotinga bartholomaii* and *Chulpasia jimthorselli*, at 3.2 kg and 0.2 kg, respectively see table 1 in Beck^37^. It may be pertinent that enlarged and procumbent (“gliriform” sensu Beck et al.^6^) lower incisors have not been reported from Tingamarra, as these are the most obvious dental synapomorphies shared by crown diprotodontians. However, the Tingamarran LF still remains poorly known, and absence of such incisors may reflect insufficient sampling. Even if additional material of *Chulpasia jimthorselli* and *Thylacotinga bartholomaii* were to confirm that they lack gliriform incisors, this would not rule out diprotodontian affinities for these two taxa, because they could be stem diprotodontians that predate the evolution of diprotodonty.

**References**

1 Cifelli, R. L. & de Muizon, C. Dentition and jaw of *Kokopellia juddi*, a primitive marsupial or near-marsupial from the medial Cretaceous of Utah. *J. Mamm. Evol.* **4**, 241–258 (1997).

2 Kielan-Jaworowska, Z., Cifelli, R. L. & Luo, Z.-X. *Mammals from the Age of Dinosaurs: Origins, Evolution, and Structure*. (Columbia University Press, 2004).

3 Davis, B. M., Cifelli, R. L. & Kielan-Jaworowska, Z. in *Mammalian Evolutionary Morphology: A Tribute to Frederick S. Szalay* (eds E J Sargis & M Dagosto) 3–24 (Springer, 2008).

4 Clemens, W. A. Fossil mammals of the Type Lance Formation, Wyoming. Part II: Marsupialia. *Univ. Calif. Publ. Geol. Sci.* **62**, 1–122 (1966).

5 Davis, B. M. A revision of 'pediomyid' marsupials from the Late Cretaceous of North America. *Acta Palaeontol. Pol.* **52** (2007).

6 Beck, R. M. D., Voss, R. S. & Jansa, S. A. Craniodental morphology and phylogeny of marsupials. *Bull. Am. Mus. Nat. Hist.* **457**, 1–352 (2022).

7 Pledge, N. S. A new species of *Muramura* Pledge (Wynyardiidae: Marsupialia) from the Middle Tertiary of the Callabonna Basin, northeastern South Australia. *Bull. Am. Mus. Nat. Hist.* **279**, 541–555 (2003).

8 Beck, R. M. D. *et al.* A bizarre new family of Marsupialia (incertae sedis) from the Early Pliocene of Northeastern Australia: implications for the phylogeny of bunodont marsupials. *J. Paleontol.* **82**, 749–762 (2008).

9 de Pinna, M. C. C. Concepts and tests of homology in the cladistic paradigm. *Cladistics* **7**, 367–394 (1991).

10 Price, G. J. & Hocknull, S. A. *Invictokoala monticola* gen. et sp. nov. (Phascolarctidae, Marsupialia), a Pleistocene plesiomorphic koala holdover from Oligocene ancestors. *J. Syst. Palaeontol.* **9**, 327–335 (2011).

11 Louys, J., Black, K., Archer, M., Hand, S. J. & Godthelp, H. Descriptions of koala fossils from the Miocene of Riversleigh, northwestern Queensland and implications for *Litokoala* (Marsupialia, Phascolarctidae). *Alcheringa* **31**, 99–110 (2007).

12 Black, K. H., Archer, M. & Hand, S. J. New Tertiary koala (Marsupialia, Phascolarctidae) from Riversleigh, Australia, with a revision of phascolarctid phylogenetics, paleoecology, and paleobiodiversity. *J. Vert. Paleontol.* **32**, 125–138 (2012).

13 Stirton, R. A., Tedford, R. H. & Woodburne, M. O. A new Tertiary formation and fauna from the Tirari Desert, South Australia. *Records of the South Australian Museum* **15**, 262–427 (1967).

14 Black, K. & Archer, M. *Nimiokoala* gen. nov. (Marsupialia, Phascolarctidae) from Riversleigh, northwestern Queensland, with a revision of *Litokoala*. *Mem. Queensl. Mus.* **41**, 209–228 (1997).

15 Black, K. H. Middle Miocene origins for tough-browse dietary specialisations in the koala (Marsupialia, Phascolarctidae) evolutionary tree: description of a new genus and species from the Riversleigh World Heritage Area. *Mem. Mus. Vic.* **74**, 255–262 (2016).

16 Sigé, B. *et al.* *Chulpasia* and *Thylacotinga*, late Paleocene-earliest Eocene trans-Antarctic Gondwanan bunodont marsupials: new data from Australia. *Geobios* **42**, 813–823 (2009).

17 Archer, M., Godthelp, H. & Hand, S. J. Early Eocene marsupial from Australia. *Kaupia* **3**, 193–200 (1993).

18 Archer, M. *Koobor notabilis* (de Vis), an unusual koala from the Pliocene Chinchilla Sand. *Mem. Queensl. Mus.* **18**, 31–35 (1977).

19 Black, K. H., Louys, J. & Price, G. J. Understanding morphological variation in the extant koala as a framework for identification of species boundaries in extinct koalas (Phascolarctidae; Marsupialia). *J. Syst. Palaeontol.* **12**, 237–264 (2013).

20 Beck, R. M. D. *et al.* A new family of diprotodontian marsupials from the latest Oligocene of Australia and the evolution of wombats, koalas, and their relatives (Vombatiformes). *Sci. Rep.* **10**, 1–13 (2020).

21 Crichton, A. I. *et al.* A new species of *Mukupirna* (Diprotodontia, Mukupirnidae) from the Oligocene of central Australia sheds light on basal vombatoid interrelationships. *Alcheringa*, doi:DOI:10.1080/03115518.2023.2181397 (2023).

22 Brewer, P., Archer, M., Hand, S. J. & Abel, R. New genus of primitive wombat (Vombatidae, Marsupialia) from Miocene deposits in the Riversleigh World Heritage area (Queensland, Australia). *Palaeontol. Electron.* **18**, 1–40 (2015).

23 Black, K. & Archer, M. *Silvabestius* gen. nov., a primitive zygomaturine (Marsupialia, Diprotodontidae) from Riversleigh, northwestern Queensland. *Mem. Queensl. Mus.* **41**, 193–208 (1997).

24 Murray, P. Primitive marsupial tapirs (*Propalorchestes novaculacephalus* Murray and *P. ponticulus* sp. nov.) from the mid-Miocene of north Australia (Marsupialia: Palorchestidae). *The Beagle: Records of the Museums and Art Galleries of the Northern Territory* **7**, 39–51 (1990).

25 Black, K. Description of new material for *Propalorchestes novaculacephalus* (Marsupialia: Palorchestidae) from the mid Miocene of Riversleigh, northwestern Queensland. *Alcheringa* **30**, 351–361 (2006).

26 Marshall, L. G., Case, J. A. & Woodburne, M. O. Phylogenetic relationships of the families of marsupials. *Current mammalogy* **2**, 433–505 (1990).

27 Horovitz, I. & Sánchez‐Villagra, M. R. A morphological analysis of marsupial mammal higher‐level phylogenetic relationships. *Cladistics* **19**, 181–212 (2003).

28 Black, K. Maradidae: a new family of vombatomorphian marsupial from the late Oligocene of Riversleigh, northwestern Queensland. *Alcheringa* **31**, 17–32 (2007).

29 Louys, J. *et al.* Cranial remains of *Ramsayia magna* from the Late Pleistocene sub-tropics of Australia and the evolution of gigantism in wombats. *Papers in Palaeontology* **8**, e1475 (2022).

30 Hope, J. H. & Wilkinson, H. E. *Warendja wakefieldi*, a new genus of wombat (Marsupialia, Vombatidae) from Pleistocene sediments in McEacherns Cave, western Victoria. *Mem. Nat. Mus. Vic.* **43**, 109–120 (1982).

31 Merrilees, D. *Cranial and mandibular characters of modern wombats (Marsupialia, Vombatidae) from a paleontological viewpoint, and their bearing on the fossils called Phascolomys parvus by Owen (1872)*. Vol. 15 (Records of the South Australian Museum, 1967).

32 Myers, T. J. Prediction of marsupial body mass. *Aust. J. Zool.* **49**, 99–118 (2001).

33 Murray, P. F. & Megirian, D. The Pwerte Marnte Marnte Local Fauna: a new vertebrate assemblage of presumed Oligocene age from the Northern Territory of Australia. *Alcheringa* **30**, 211–228 (2006).

34 Travouillon, K. J., Beck, R. M. D. & Case, J. A. Upper Oligocene–lower-Middle Miocene peramelemorphians from the Etadunna, Namba and Wipajiri formations of South Australia. *Alcheringa* **45**, 109-125 (2021).

35 Beck, R. M. D., Godthelp, H., Weisbecker, V., Archer, M. & Hand, S. J. Australia's oldest marsupial fossils and their biogeographical implications. *PLoS One* **3**, e1858 (2008).

36 Kealy, S. & Beck, R. Total evidence phylogeny and evolutionary timescale for Australian faunivorous marsupials (Dasyuromorphia). *BMC Evol. Biol.* **17**, 1–23 (2017).

37 Beck, R. M. D. A peculiar faunivorous metatherian from the Early Eocene of Australia. *Acta Palaeontol. Pol.* **60**, 123–129 (2013).
